# Supplementary material for: Snf1 and yeast GSK3-β activates Tda1 to suppress glucose starvation signaling
Source: EMBO Rep. 2025 Apr 24;26(11):2910–30. doi: 10.1038/s44319-025-00456-y (PMC12152124; doi:10.1038/s44319-025-00456-y)
Supplement: Supplementary file 1 — Appendix [file 44319_2025_456_MOESM1_ESM.pdf]

Table of content

Appendix Fig. S1	2
Appendix Fig. S2	3
Appendix Fig. S3	4
Appendix Fig. S4	5
Appendix Fig. S5	6
Appendix Table S1	7
Appendix Table S2	8

Appendix Fig. S1

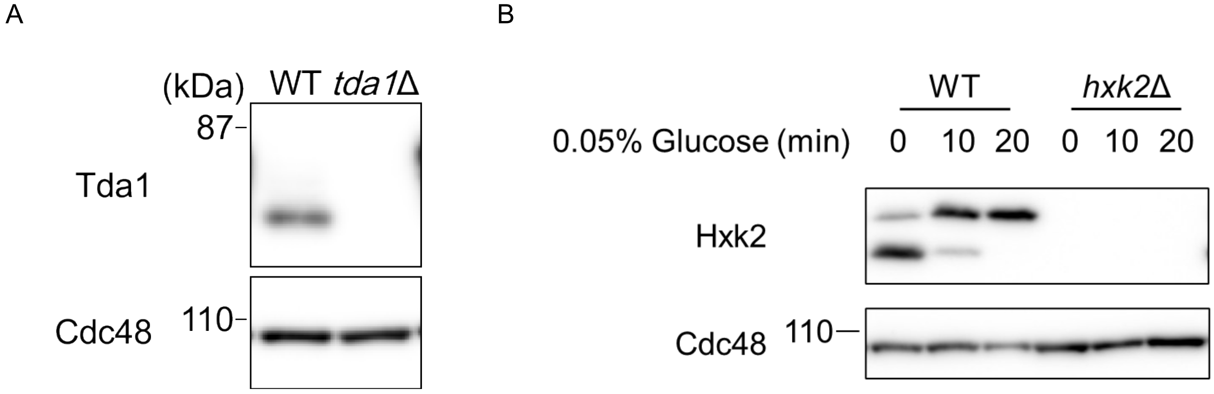

Appendix Figure S1. Anti-Tda1 and anti-Hxk2 antibodies specifically detect Tda1 and Hxk2, respectively.
(A) The indicated strains were grown to log phase in YPD medium. Total cell lysates were prepared using the TCA lysis method and analyzed by immunoblotting with anti-Tda1 and anti-Cdc48. **(B)** The indicated strains were grown to log phase in YPD medium. Subsequently, the medium was switched from YPD to YP with 0.05% glucose, and cells were harvested at the indicated time points. Total cell lysates were resolved by Phos-tag SDS-PAGE and analyzed via immunoblotting using anti-Hxk2 and anti-Cdc48.

Appendix Fig. S2

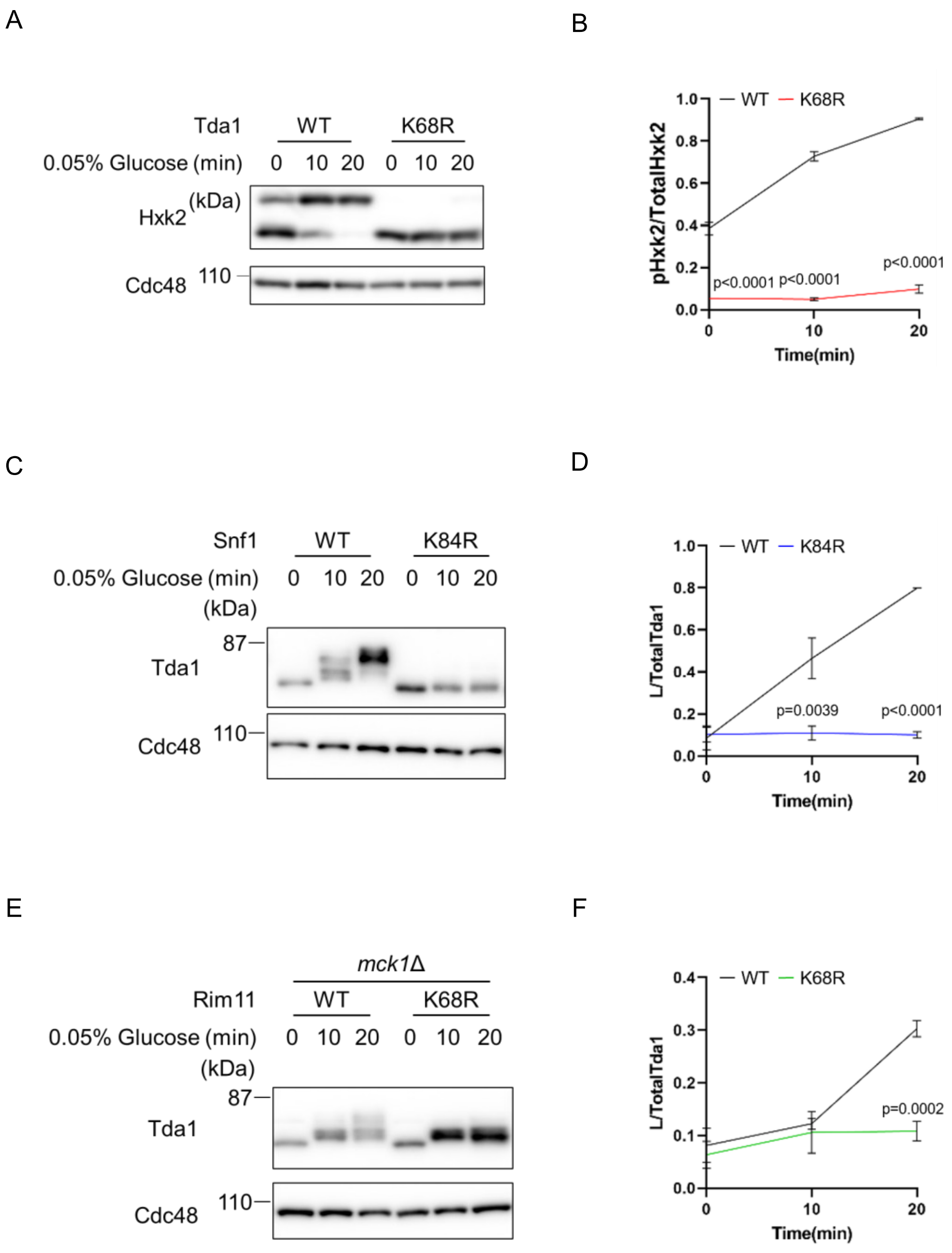

Appendix Figure S2. Kinase-dead mutants of Tda1, Snf1, and Rim11 show decreased phosphorylation activity toward their target proteins.

(A, C, E) Lysates from the indicated strains were prepared and analyzed via immunoblotting as described in Appendix Figure S1B. **(B)** The relative levels of phosphorylated Hxk2 to total Hxk2 were quantified. Error bars indicate the standard deviation (SD) of three independent experiments. p-value was calculated by unpaired two-tailed t test. **(D, F)** The relative levels of low mobility Tda1 to total Tda1 were quantified as described in Fig. 1C. Error bars indicate the standard deviation (SD) of three independent experiments. p-value was calculated by unpaired two-tailed t test.

Appendix Fig. S3

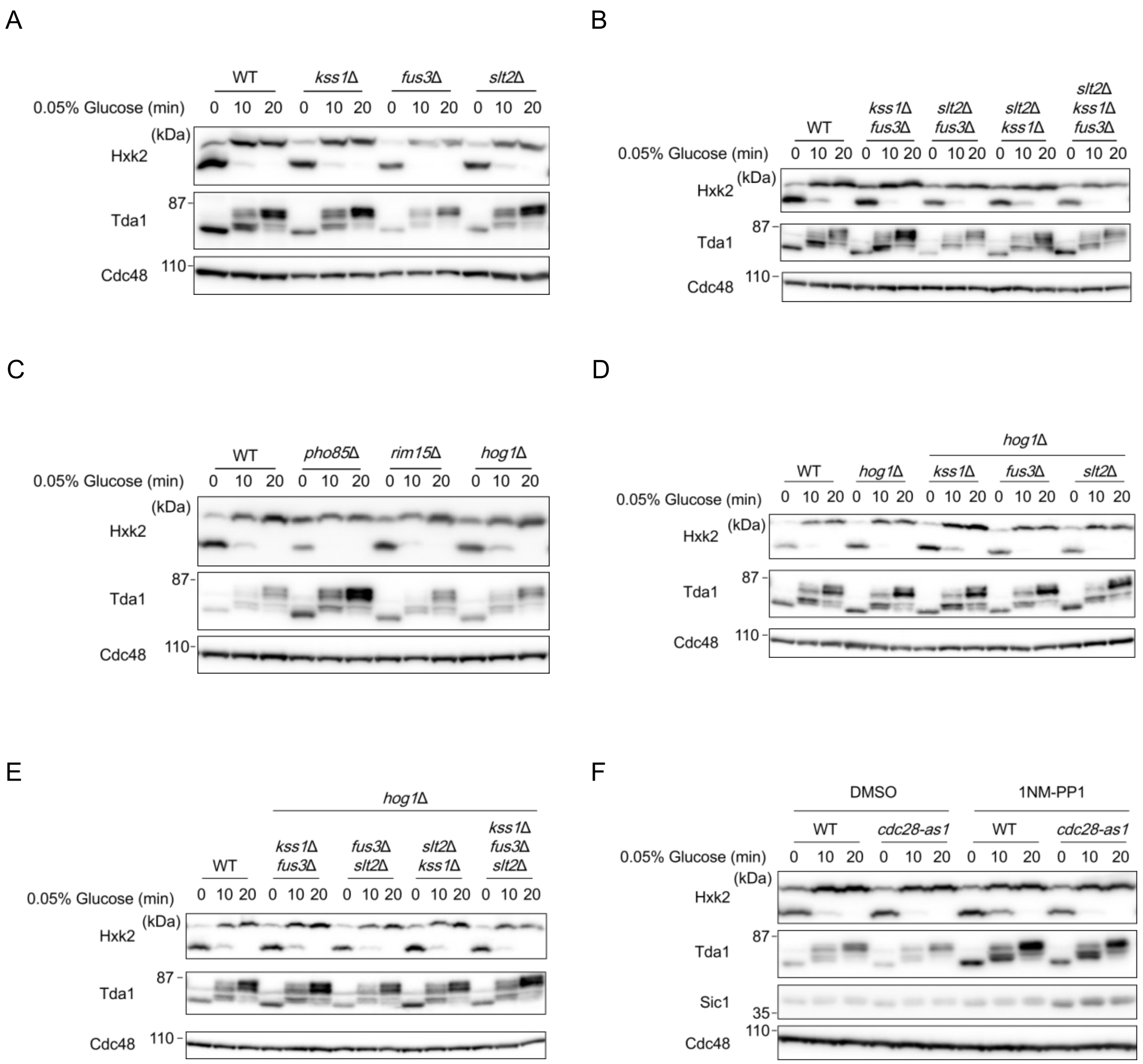

Appendix Figure S3. Identification of kinases responsible for the phosphorylation of Tda1 at Thr513.

(A-E) Lysates from the indicated strains were prepared and analyzed via immunoblotting as described in Appendix Figure S1B. Total cell lysates were resolved by Phos-tag SDS-PAGE (to detect Hxk2) or SDS-PAGE (to detect Tda1 and Cdc48) and analyzed via immunoblotting using anti-Hxk2 (**A-E**), anti-Tda1 (**A-E**), and anti-Cdc48 (**A-E**). **(F)** The indicated strains were grown to log phase in YPD medium, followed by incubation with DMSO or 1NM-PP1 for 30 minutes. After shifting to YP medium with 0.05% glucose, samples were collected at the specified time points. Total cell lysates were resolved by Phos-tag SDS-PAGE (to detect Hxk2) or SDS-PAGE (to detect Tda1 and Cdc48) and analyzed via immunoblotting using anti-Hxk2, anti-Tda1, anti-Sic1, and anti-Cdc48.

Appendix Fig. S4

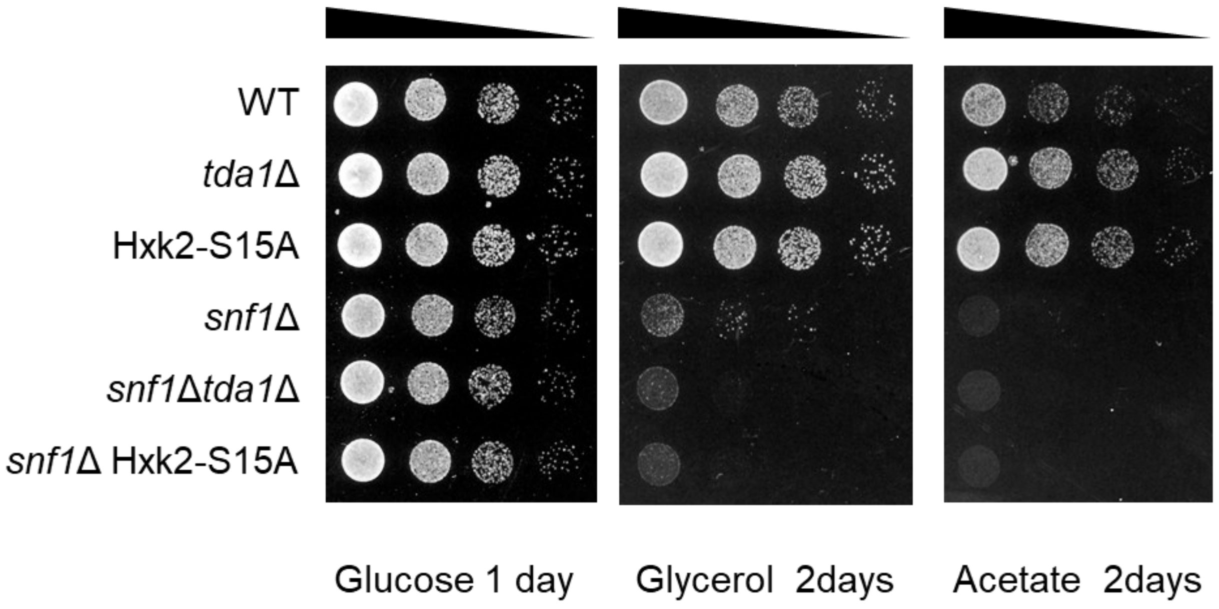

Appendix Figure S4. Snf1 is required for growth promotion of Hxk2-S15A and *tda1*Δ mutants on glycerol plates.

The indicated strains were grown to log phase, adjusted to a cell density of $OD_{600} = 0.3$, serially diluted 1:10, spotted onto plates containing the specified carbon sources, and incubated at 30˚C for the indicated durations.

Appendix Fig. S5

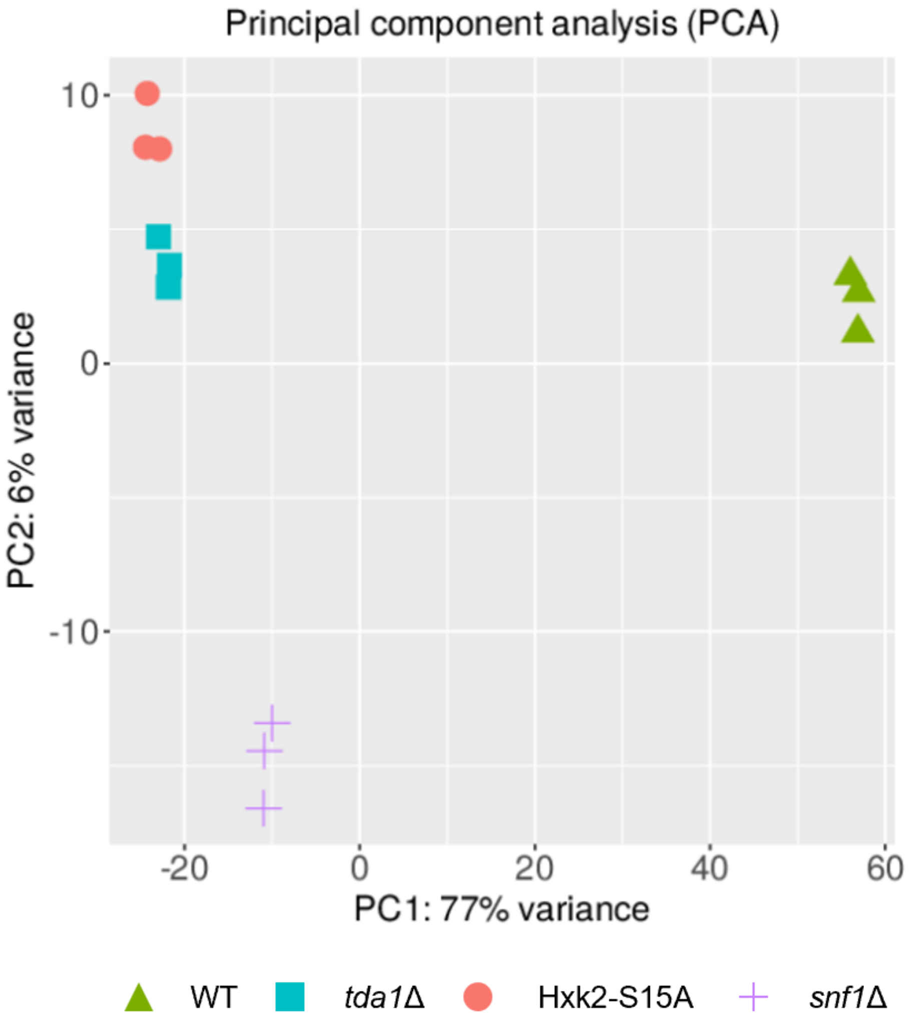

Appendix Figure S5. Principal component analysis of RNA-seq
Principal component analysis was performed on the RNA-seq data presented in Fig. 6B. The plots were created by iDEP v.0.96.

Appendix Table S1. Kinases co-purified with Tda1-3xHA

Proteins annotated as Kinase were listed. Scores are also listed.

Protein	Accession	Description	Score Sequest HT
Rim11	P38615	Serine/threonine-protein kinase RIM11/MSD1	58.69
Snf1	P06782	Carbon catabolite-derepressing protein kinase	42.23
Hog1	P32485	Mitogen-activated protein kinase HOG1	22.04
Pbs2	P08018	MAP kinase kinase PBS2	19.92
Ypk1	P12688	Serine/threonine-protein kinase YPK1	14.31
Hrr25	P29295	Casein kinase I homolog HRR25	7.88
Hrk1	Q08732	Serine/threonine-protein kinase HRK1	7.57
Cdc28	P00546	Cyclin-dependent kinase 1	7.37
Ste20	Q03497	Serine/threonine-protein kinase STE20	4.6
Pho85	P17157	Cyclin-dependent protein kinase PHO85	4.57
Cla4	P48562	Serine/threonine-protein kinase CLA4	4.23
Kin2	P13186	Serine/threonine-protein kinase KIN2	3.69
Fus3	P16892	Mitogen-activated protein kinase FUS3	3.55
Slt2	Q00772	Mitogen-activated protein kinase SLT2/MPK1	3.52
Smk1	P41808	Sporulation-specific mitogen-activated protein kinase SMK1	3.52
Tor2	P32600	Serine/threonine-protein kinase TOR2	3.46
Sch9	P11792	Serine/threonine-protein kinase SCH9	3.3
Ypk2	P18961	Serine/threonine-protein kinase YPK2/YKR2	3.27
Psk2	Q08217	Serine/threonine-protein kinase PSK2	2.78
Hal5	P38970	Serine/threonine-protein kinase HAL5	2.13
Mck1	P21965	Protein kinase MCK1	2.03
Mrk1	P50873	Serine/threonine-protein kinase MRK1	2.03
Mkk2	P32491	MAP kinase kinase MKK2/SSP33	2.03
Kss1	P14681	Mitogen-activated protein kinase KSS1	1.86
Psk1	P31374	Serine/threonine-protein kinase PSK1	1.73

Score is calculated by summing the individual scores of each peptide. Sequest HT is the name of the employed search engine.

Appendix Table S2. Raw paired-end sequencing reads statistics.

Name	Number of sequences	Sum of length	Average length
WT_Glycerol6h_rep1_R1.fastq.gz	4,338,288	351,401,328	81
WT_Glycerol6h_rep1_R2.fastq.gz	4,338,288	351,401,328	81
WT_Glycerol6h_rep2_R1.fastq.gz	4,652,288	376,835,328	81
WT_Glycerol6h_rep2_R2.fastq.gz	4,652,288	376,835,328	81
WT_Glycerol6h_rep3_R1.fastq.gz	5,390,480	436,628,880	81
WT_Glycerol6h_rep3_R2.fastq.gz	5,390,480	436,628,880	81
tda1deletion_Glycerol6h_rep1_R1.fastq.gz	5,974,049	483,897,969	81
tda1deletion_Glycerol6h_rep1_R2.fastq.gz	5,974,049	483,897,969	81
tda1deletion_Glycerol6h_rep2_R1.fastq.gz	5,239,692	424,415,052	81
tda1deletion_Glycerol6h_rep2_R2.fastq.gz	5,239,692	424,415,052	81
tda1deletion_Glycerol6h_rep3_R1.fastq.gz	5,836,009	472,716,729	81
tda1deletion_Glycerol6h_rep3_R2.fastq.gz	5,836,009	472,716,729	81
snf1deletion_Glycerol6h_rep1_R1.fastq.gz	5,415,711	438,672,591	81
snf1deletion_Glycerol6h_rep1_R2.fastq.gz	5,415,711	438,672,591	81
snf1deletion_Glycerol6h_rep2_R1.fastq.gz	5,318,360	430,787,160	81
snf1deletion_Glycerol6h_rep2_R2.fastq.gz	5,318,360	430,787,160	81
snf1deletion_Glycerol6h_rep3_R1.fastq.gz	5,290,411	428,523,291	81
snf1deletion_Glycerol6h_rep3_R2.fastq.gz	5,290,411	428,523,291	81
Hxk2-S15A_Glycerol6h_rep1_R1.fastq.gz	4,250,462	344,287,422	81
Hxk2-S15A_Glycerol6h_rep1_R2.fastq.gz	4,250,462	344,287,422	81
Hxk2-S15A_Glycerol6h_rep2_R1.fastq.gz	4,448,359	360,317,079	81
Hxk2-S15A_Glycerol6h_rep2_R2.fastq.gz	4,448,359	360,317,079	81
Hxk2-S15A_Glycerol6h_rep3_R1.fastq.gz	4,383,700	355,079,700	81
Hxk2-S15A_Glycerol6h_rep3_R2.fastq.gz	4,383,700	355,079,700	81
