# Supplementary figures and images for: Snf1 and yeast GSK3-β activates Tda1 to suppress glucose starvation signaling
Source: EMBO Rep. 2025 Apr 24;26(11):2910–30. doi: 10.1038/s44319-025-00456-y (PMC12152124; doi:10.1038/s44319-025-00456-y)

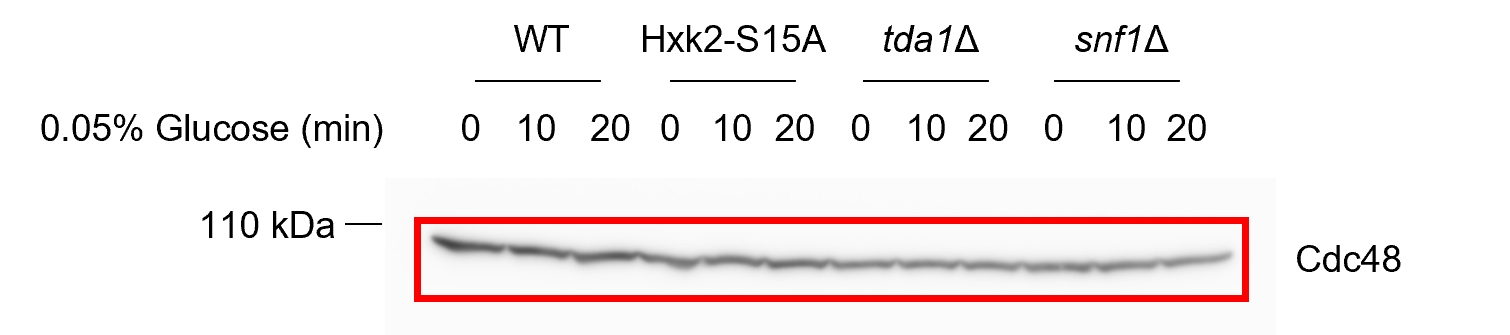

Supplement: Supplementary file 5 — Source data Fig. 1 [file 44319_2025_456_MOESM5_ESM.zip › Figure 1/Figure 1A/Figure 1A Cdc48.tif]

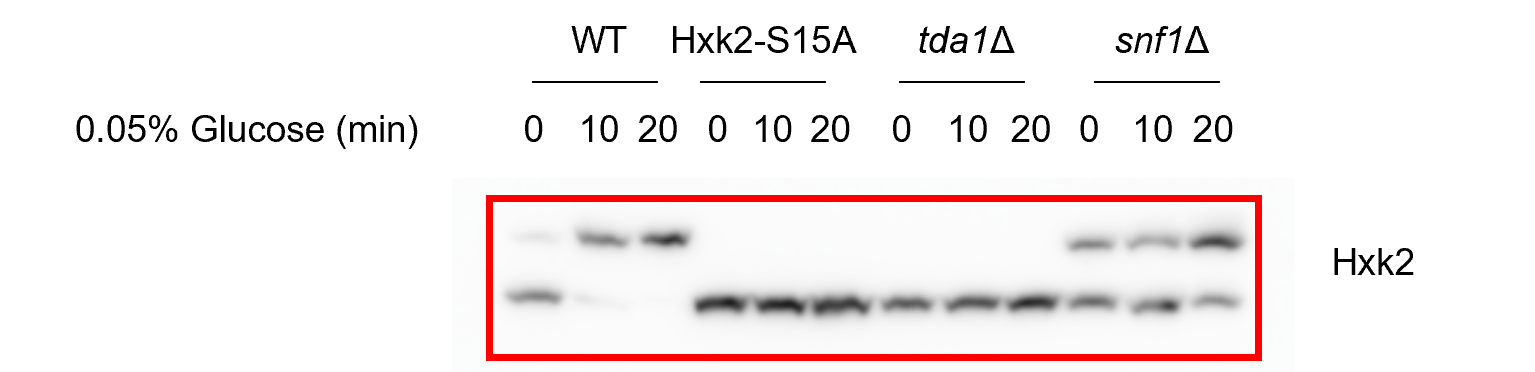

Supplement: Supplementary file 5 — Source data Fig. 1 [file 44319_2025_456_MOESM5_ESM.zip › Figure 1/Figure 1A/Figure 1A Hxk2.tif]

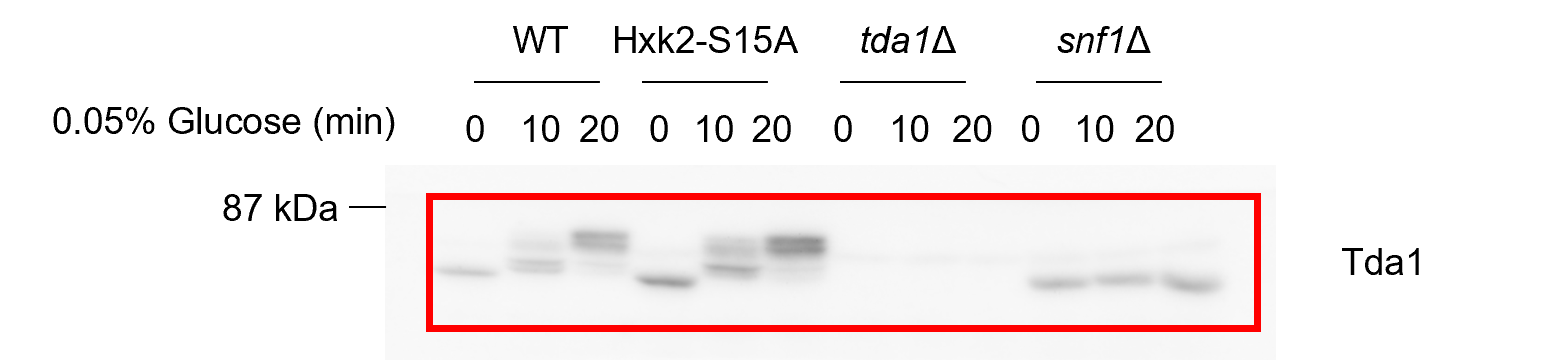

Supplement: Supplementary file 5 — Source data Fig. 1 [file 44319_2025_456_MOESM5_ESM.zip › Figure 1/Figure 1A/Figure 1A Tda1.tif]

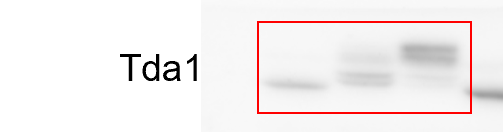

Supplement: Supplementary file 5 — Source data Fig. 1 [file 44319_2025_456_MOESM5_ESM.zip › Figure 1/Figure 1C/Figure 1C.tif]

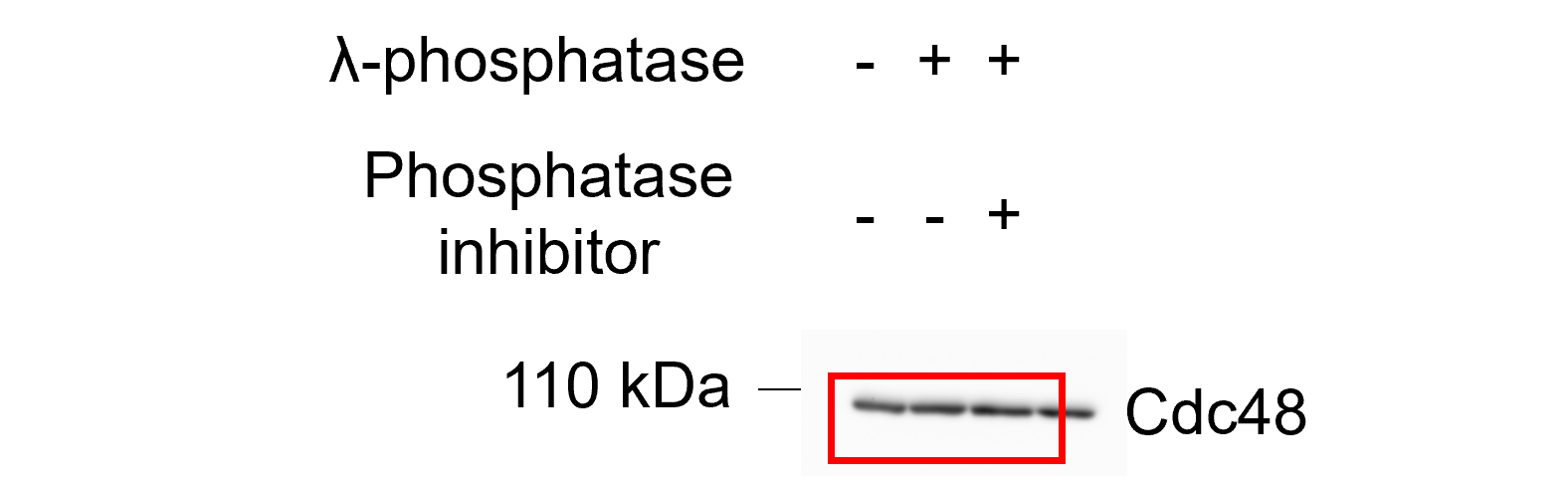

Supplement: Supplementary file 5 — Source data Fig. 1 [file 44319_2025_456_MOESM5_ESM.zip › Figure 1/Figure 1D/Figure 1D Cdc48.tif]

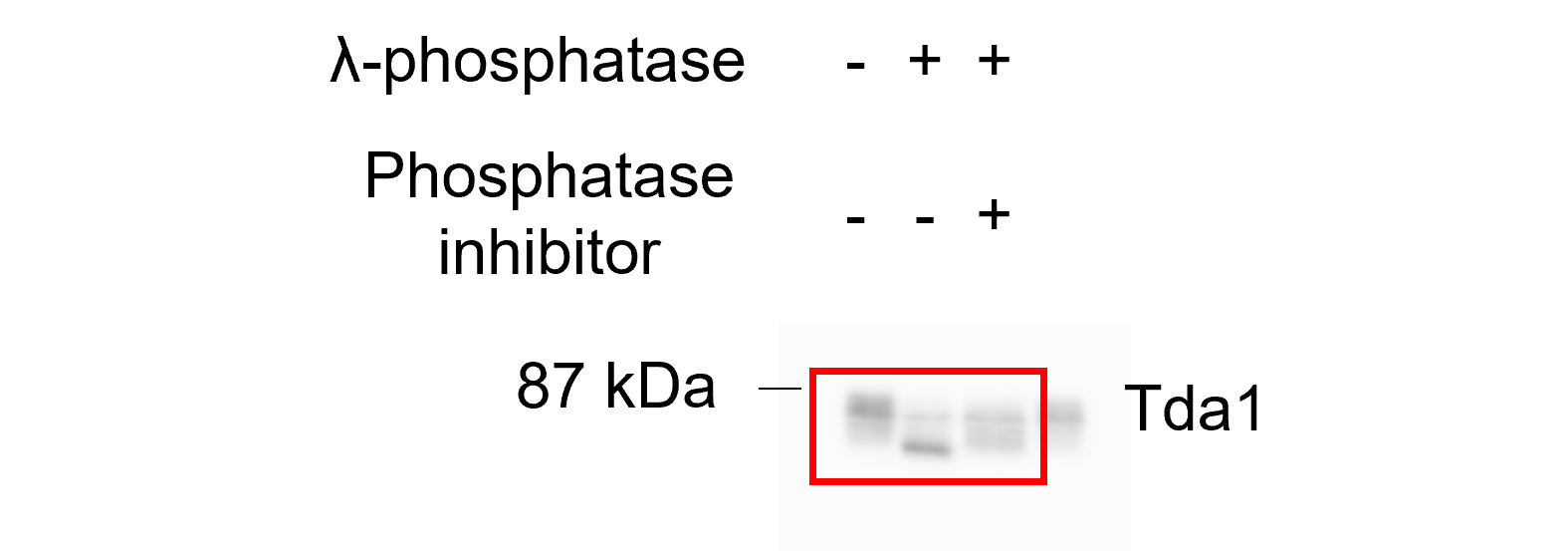

Supplement: Supplementary file 5 — Source data Fig. 1 [file 44319_2025_456_MOESM5_ESM.zip › Figure 1/Figure 1D/Figure 1D Tda1.tif]

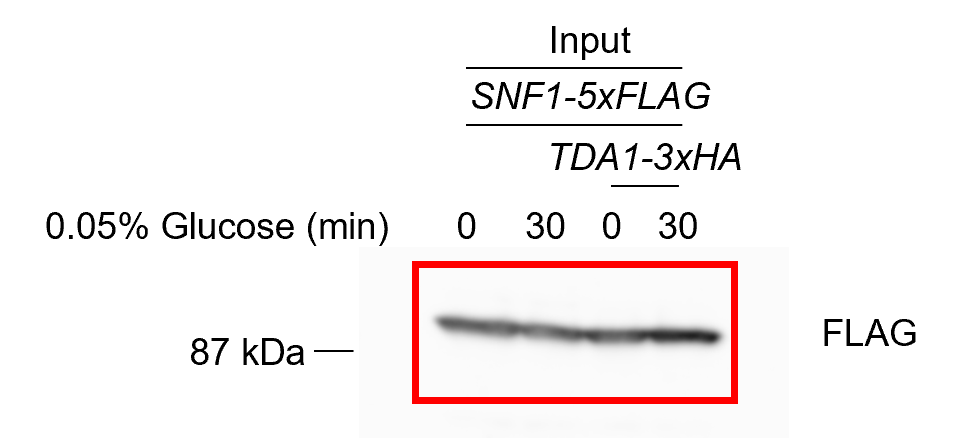

Supplement: Supplementary file 5 — Source data Fig. 1 [file 44319_2025_456_MOESM5_ESM.zip › Figure 1/Figure 1E/Figure 1E Input FLAG.tif]

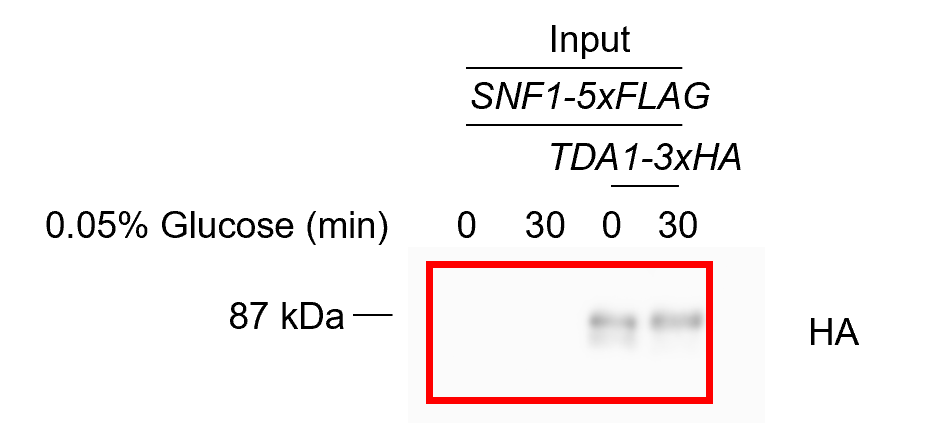

Supplement: Supplementary file 5 — Source data Fig. 1 [file 44319_2025_456_MOESM5_ESM.zip › Figure 1/Figure 1E/Figure 1E Input HA.tif]

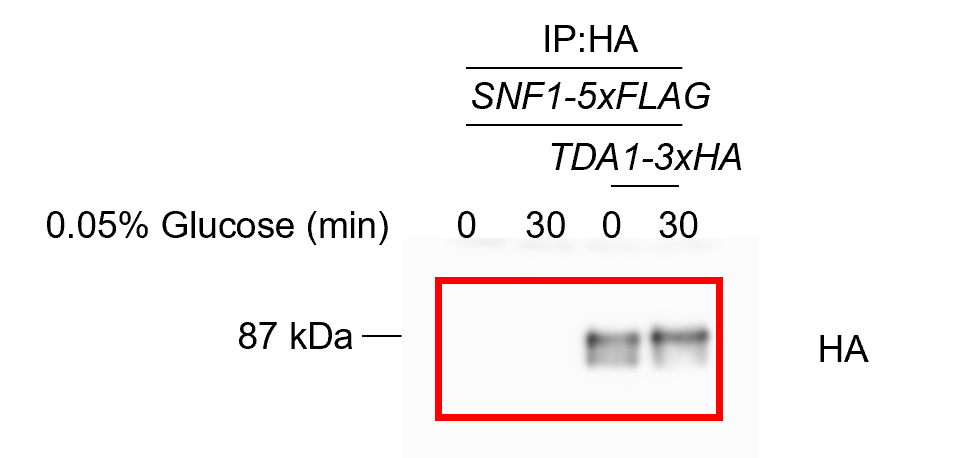

Supplement: Supplementary file 5 — Source data Fig. 1 [file 44319_2025_456_MOESM5_ESM.zip › Figure 1/Figure 1E/Figure 1E IP HA.tif]

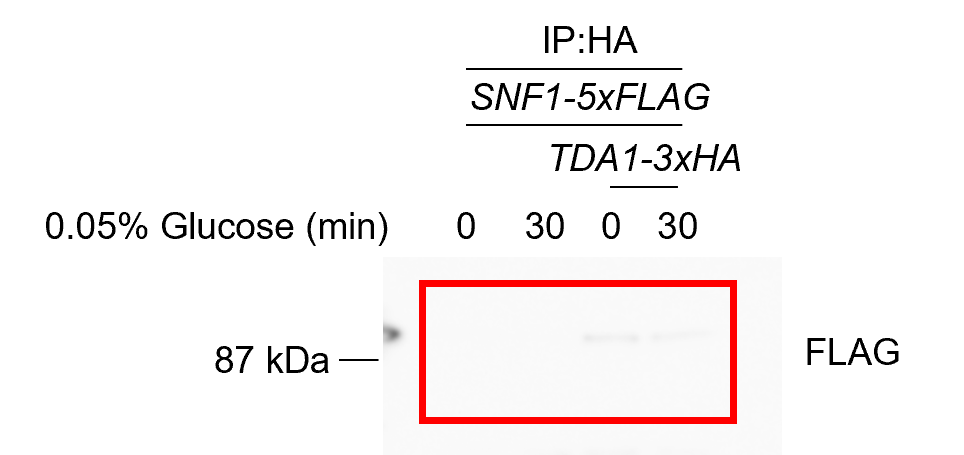

Supplement: Supplementary file 5 — Source data Fig. 1 [file 44319_2025_456_MOESM5_ESM.zip › Figure 1/Figure 1E/Figure 1E IPvFLAG.tif]

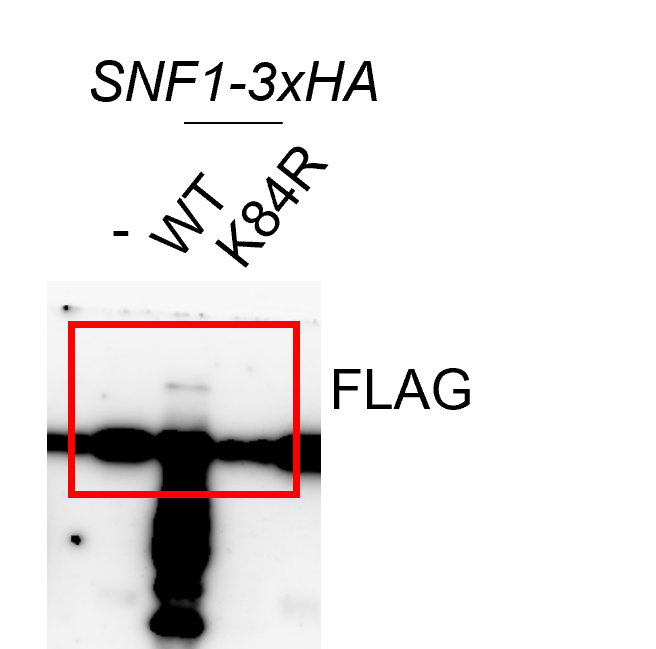

Supplement: Supplementary file 5 — Source data Fig. 1 [file 44319_2025_456_MOESM5_ESM.zip › Figure 1/Figure 1F/Figure 1F FLAG.tif]

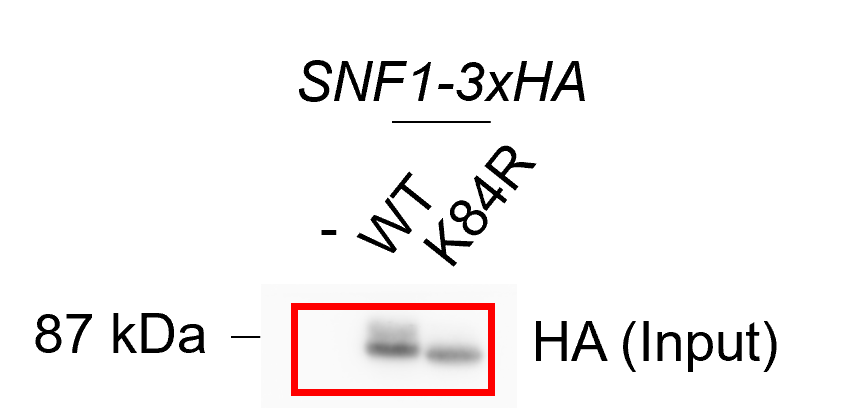

Supplement: Supplementary file 5 — Source data Fig. 1 [file 44319_2025_456_MOESM5_ESM.zip › Figure 1/Figure 1F/Figure 1F HA Input.tif]

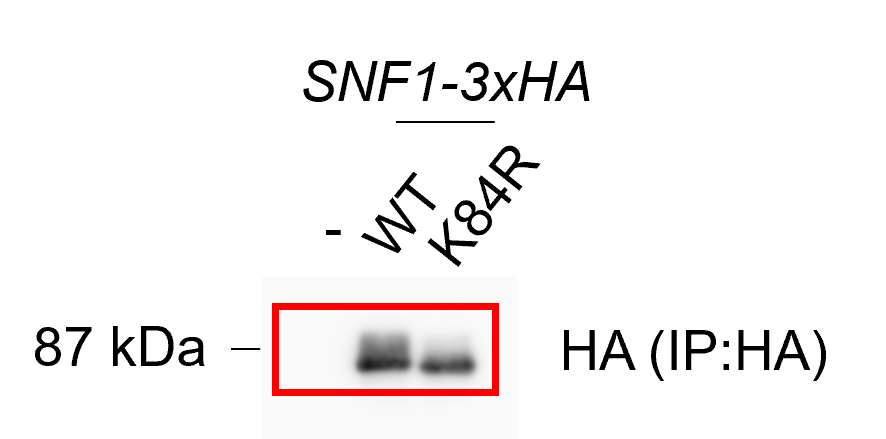

Supplement: Supplementary file 5 — Source data Fig. 1 [file 44319_2025_456_MOESM5_ESM.zip › Figure 1/Figure 1F/Figure 1F HA IP.tif]

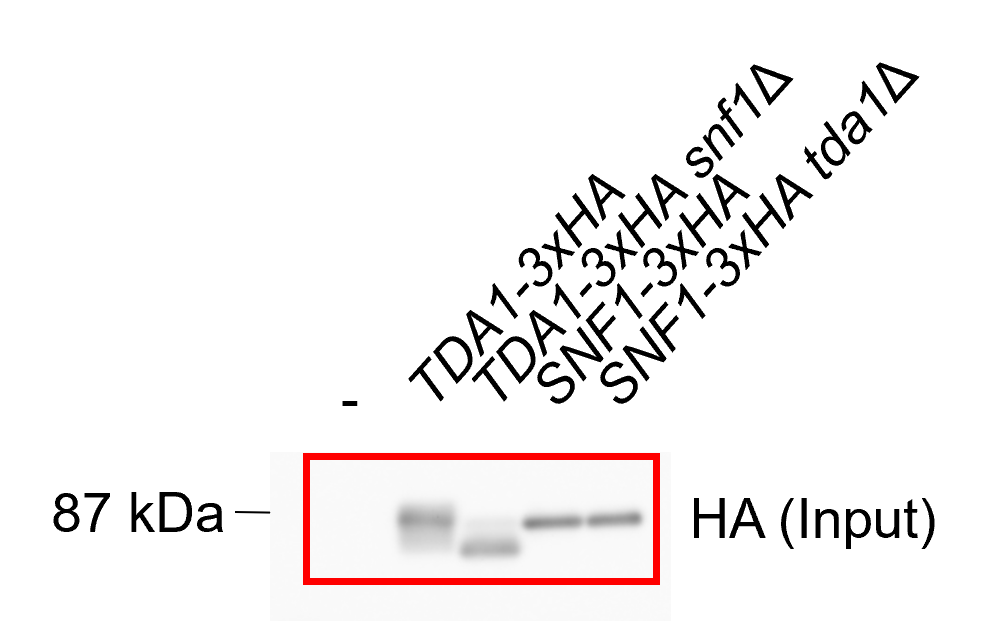

Supplement: Supplementary file 5 — Source data Fig. 1 [file 44319_2025_456_MOESM5_ESM.zip › Figure 1/Figure 1G/Figure 1G HA Input.tif]

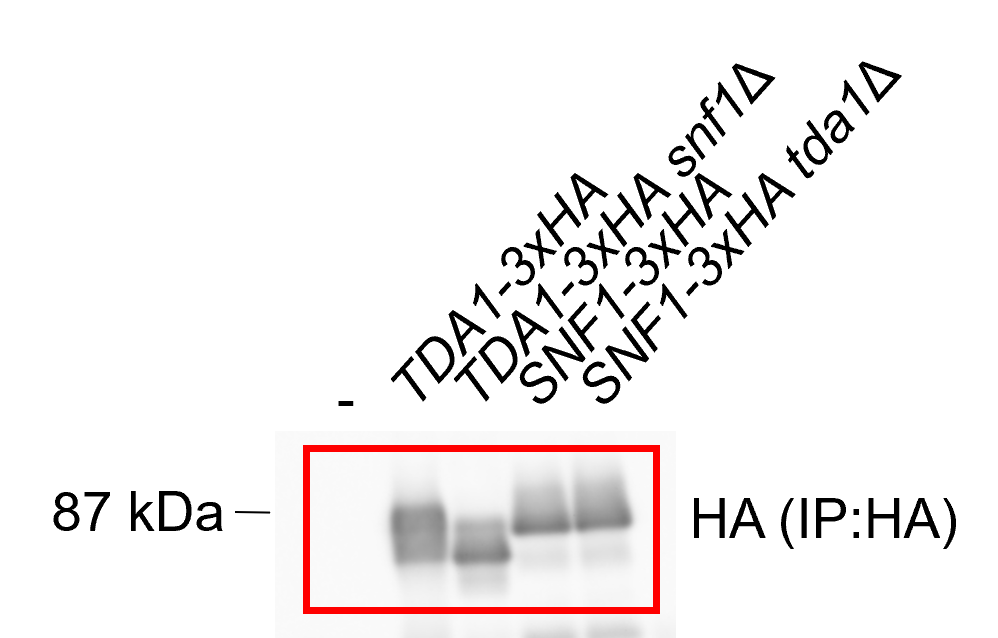

Supplement: Supplementary file 5 — Source data Fig. 1 [file 44319_2025_456_MOESM5_ESM.zip › Figure 1/Figure 1G/Figure 1G HA IP.tif]

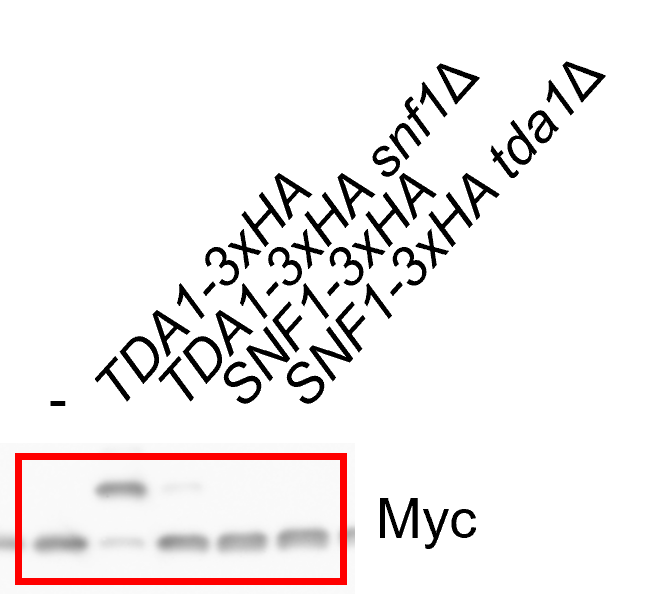

Supplement: Supplementary file 5 — Source data Fig. 1 [file 44319_2025_456_MOESM5_ESM.zip › Figure 1/Figure 1G/Figure 1G Myc.tif]

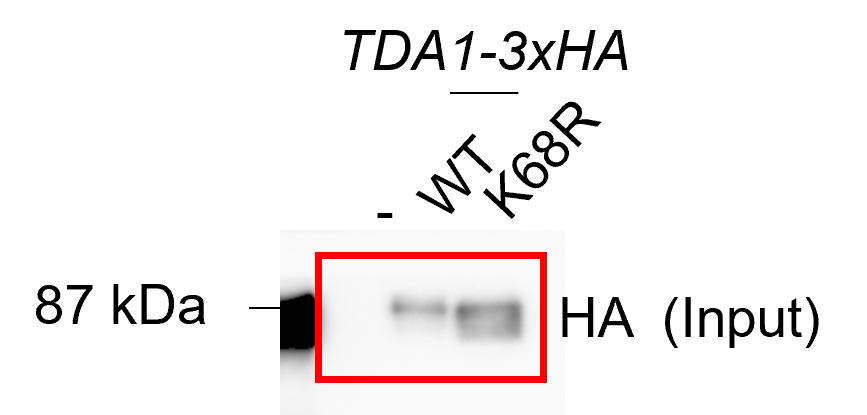

Supplement: Supplementary file 5 — Source data Fig. 1 [file 44319_2025_456_MOESM5_ESM.zip › Figure 1/Figure 1H/Figure 1H HA Input.tif]

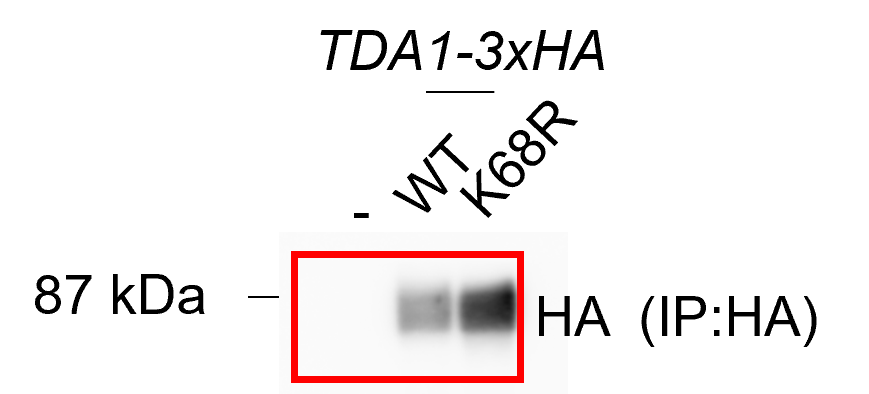

Supplement: Supplementary file 5 — Source data Fig. 1 [file 44319_2025_456_MOESM5_ESM.zip › Figure 1/Figure 1H/Figure 1H HA IP.tif]

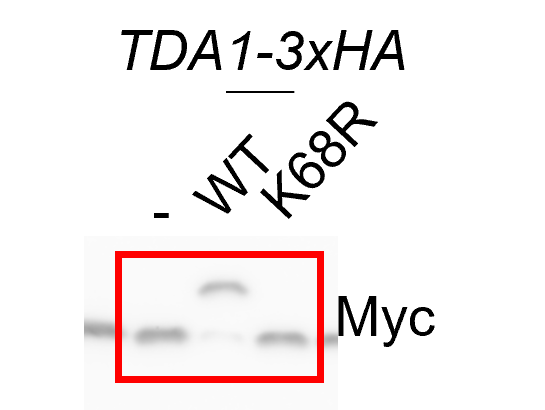

Supplement: Supplementary file 5 — Source data Fig. 1 [file 44319_2025_456_MOESM5_ESM.zip › Figure 1/Figure 1H/Figure 1H Myc.tif]

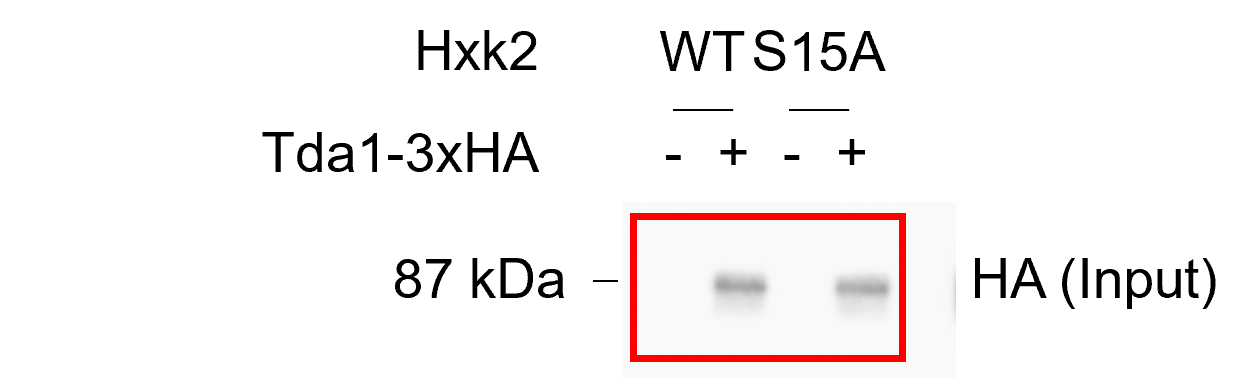

Supplement: Supplementary file 5 — Source data Fig. 1 [file 44319_2025_456_MOESM5_ESM.zip › Figure 1/Figure 1I/Figure 1I HA Input.tif]

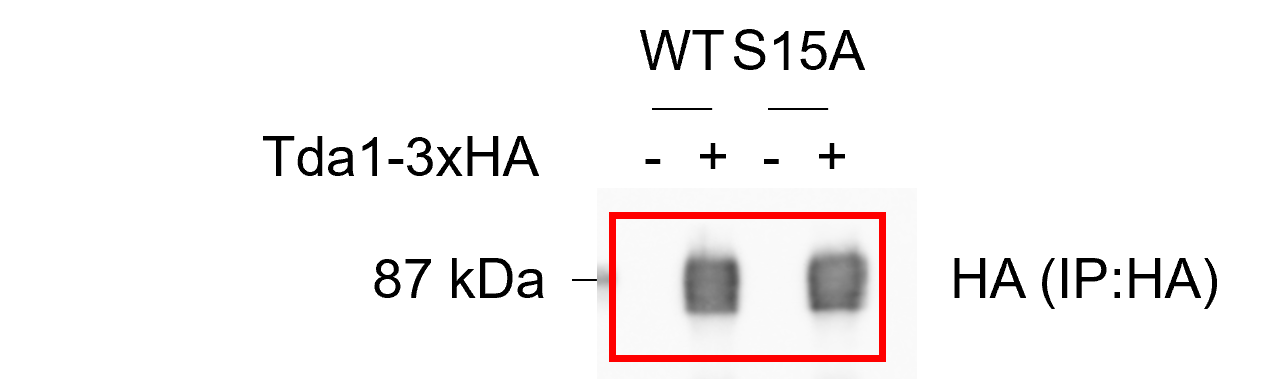

Supplement: Supplementary file 5 — Source data Fig. 1 [file 44319_2025_456_MOESM5_ESM.zip › Figure 1/Figure 1I/Figure 1I HA IP.tif]

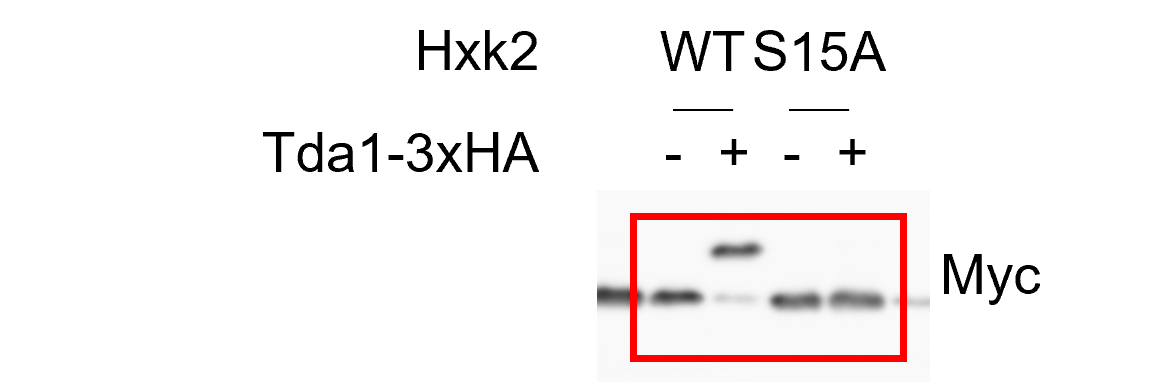

Supplement: Supplementary file 5 — Source data Fig. 1 [file 44319_2025_456_MOESM5_ESM.zip › Figure 1/Figure 1I/Figure 1I Myc.tif]

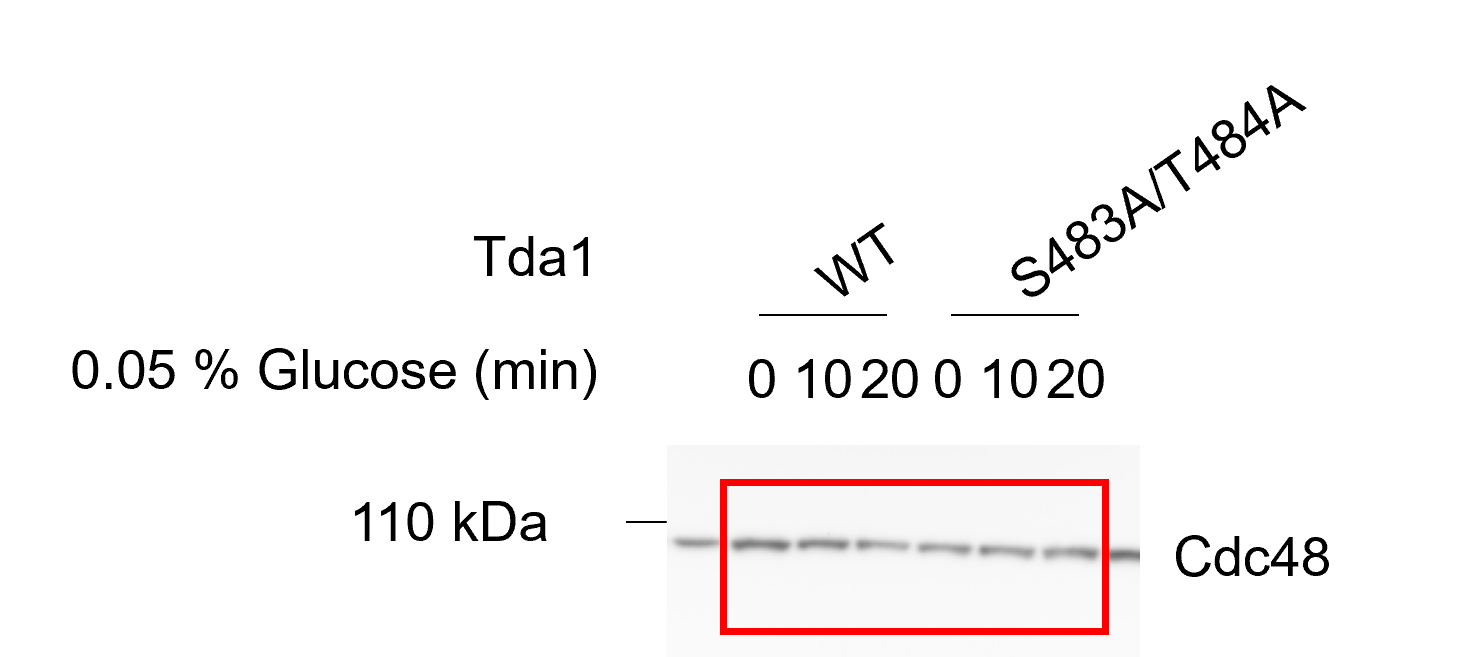

Supplement: Supplementary file 6 — Source data Fig. 2 [file 44319_2025_456_MOESM6_ESM.zip › Figure 2/Figure 2A/Figure 2A Cdc48.tif]

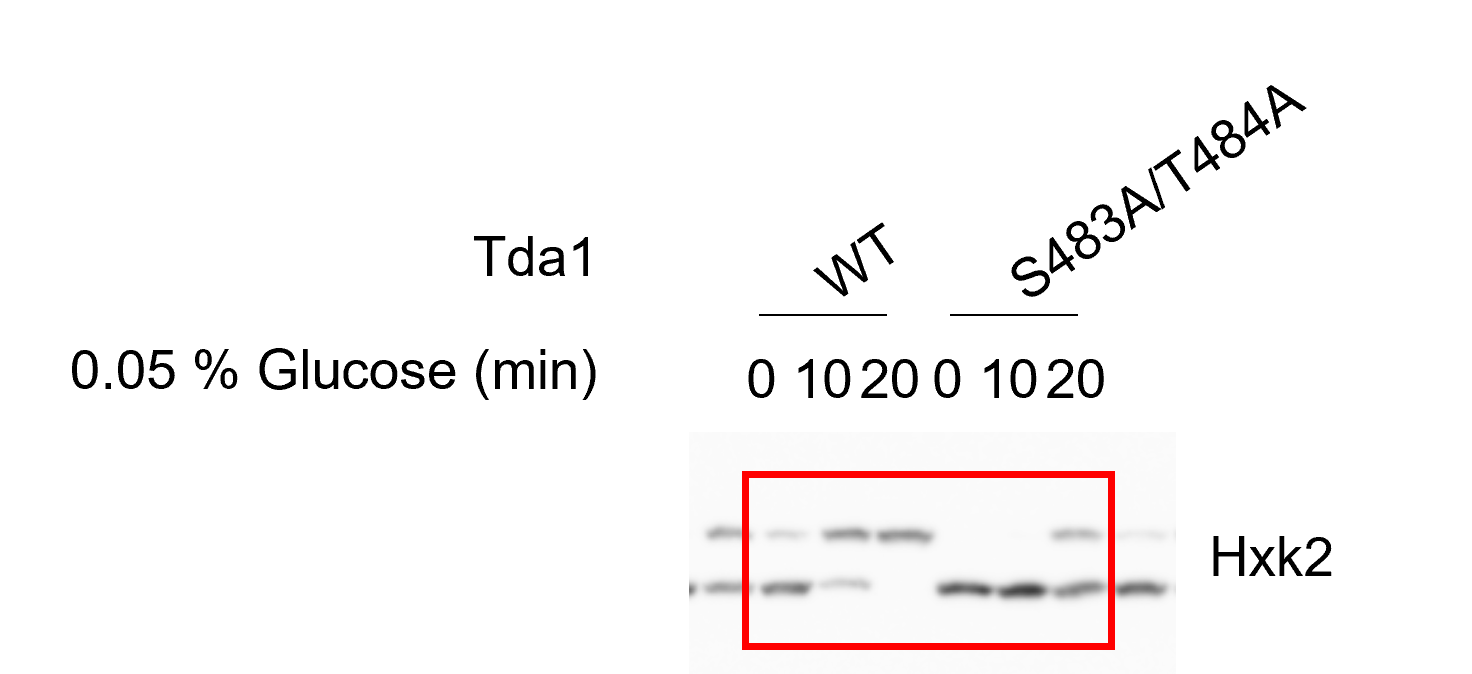

Supplement: Supplementary file 6 — Source data Fig. 2 [file 44319_2025_456_MOESM6_ESM.zip › Figure 2/Figure 2A/Figure 2A Hxk2.tif]

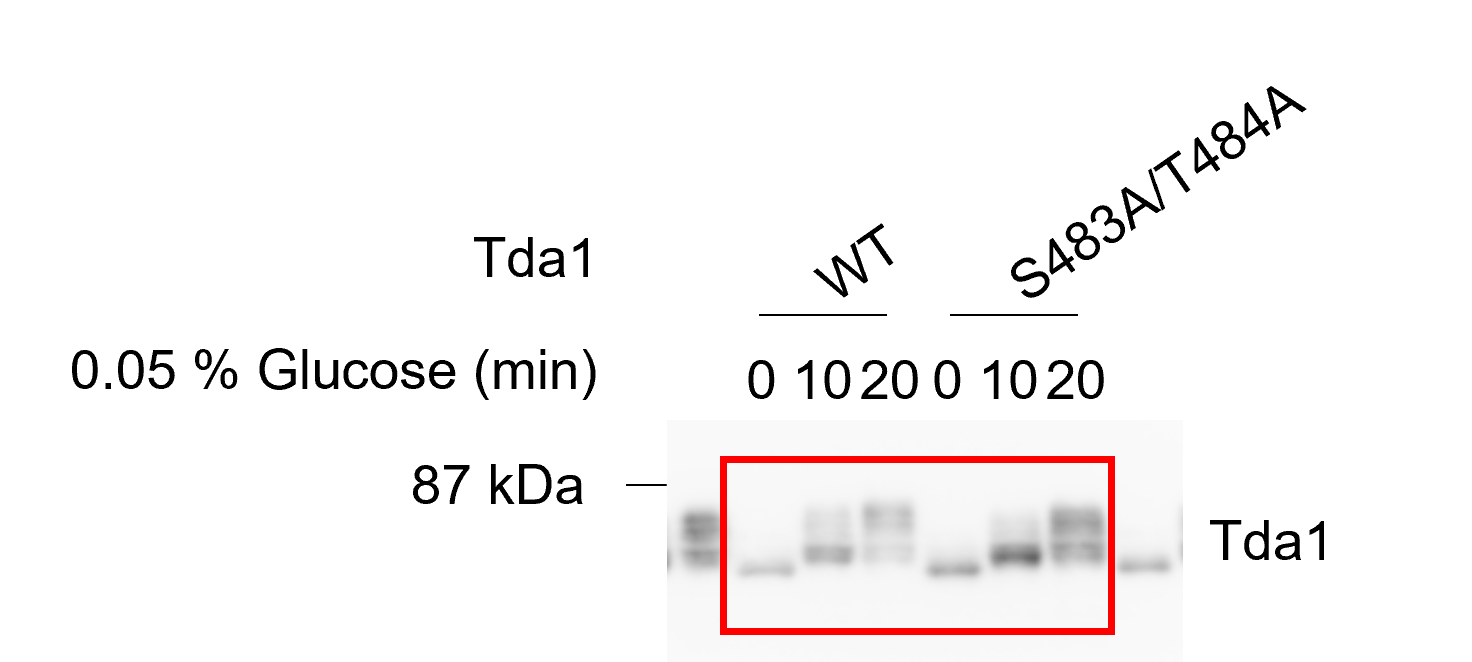

Supplement: Supplementary file 6 — Source data Fig. 2 [file 44319_2025_456_MOESM6_ESM.zip › Figure 2/Figure 2A/Figure 2A Tda1.tif]

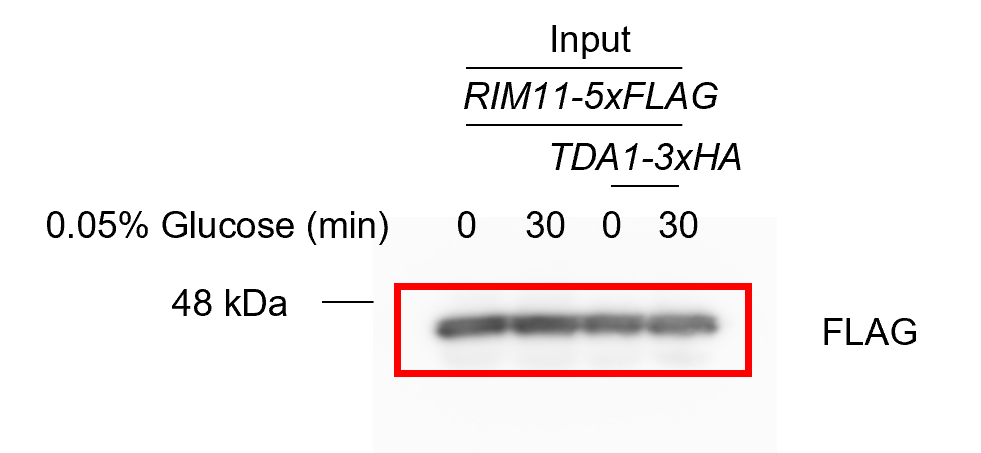

Supplement: Supplementary file 7 — Source data Fig. 3 [file 44319_2025_456_MOESM7_ESM.zip › Figure 3/Figure 3A/Figure 3A FLAG Input.tif]

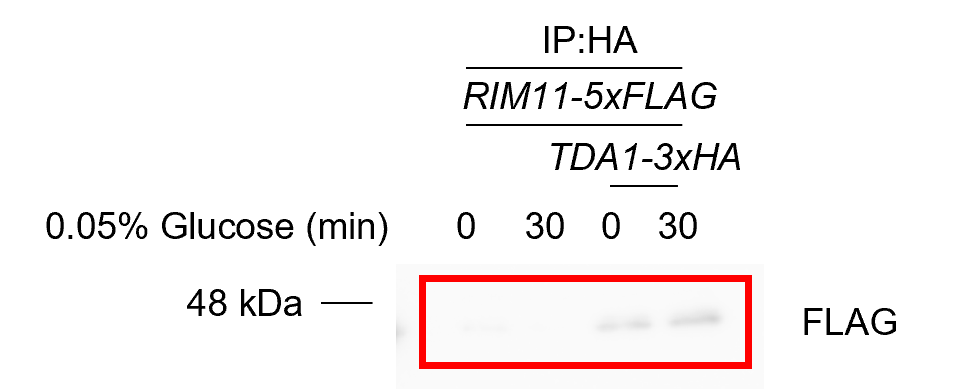

Supplement: Supplementary file 7 — Source data Fig. 3 [file 44319_2025_456_MOESM7_ESM.zip › Figure 3/Figure 3A/Figure 3A FLAG IP.tif]

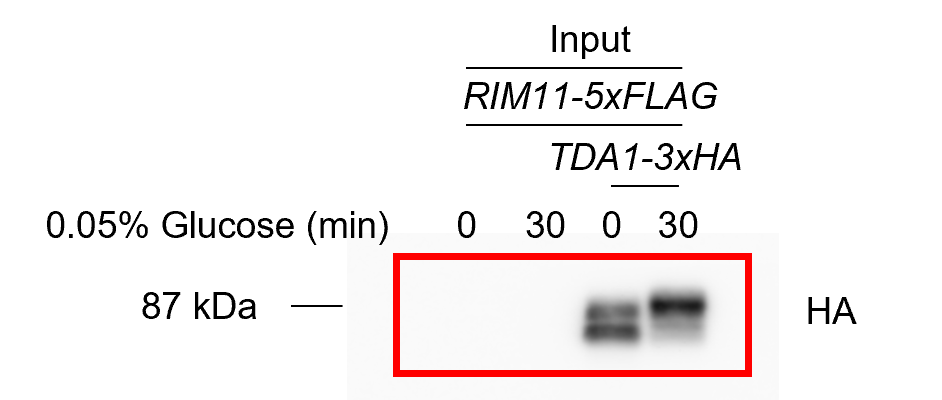

Supplement: Supplementary file 7 — Source data Fig. 3 [file 44319_2025_456_MOESM7_ESM.zip › Figure 3/Figure 3A/Figure 3A HA Input.tif]

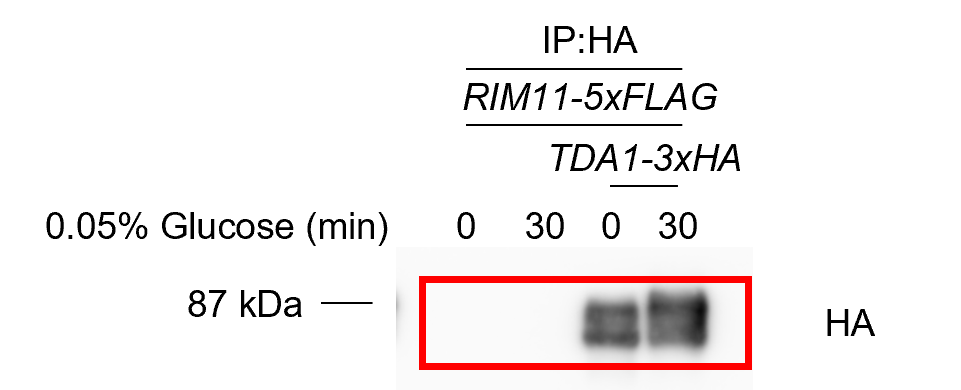

Supplement: Supplementary file 7 — Source data Fig. 3 [file 44319_2025_456_MOESM7_ESM.zip › Figure 3/Figure 3A/Figure 3A HA IP.tif]

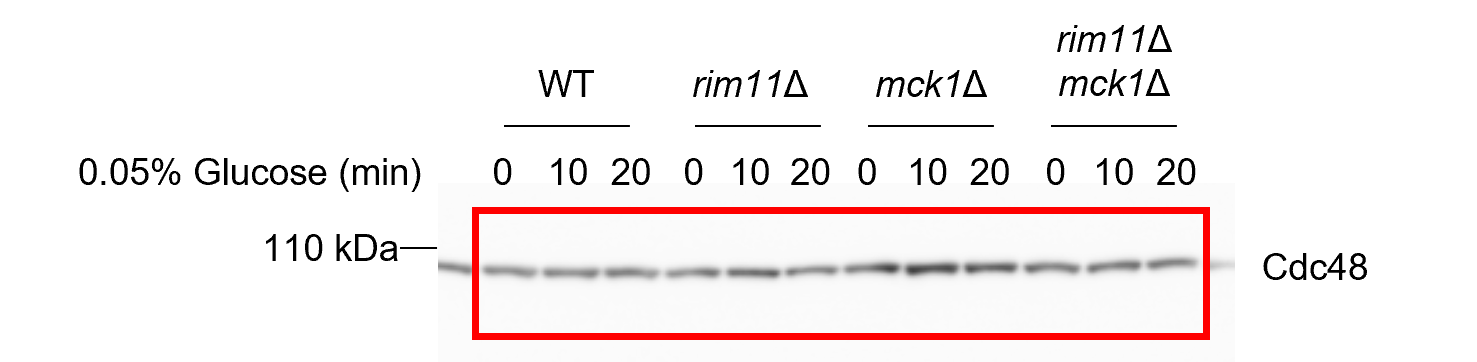

Supplement: Supplementary file 7 — Source data Fig. 3 [file 44319_2025_456_MOESM7_ESM.zip › Figure 3/Figure 3B/Figure 3B Cdc48.tif]

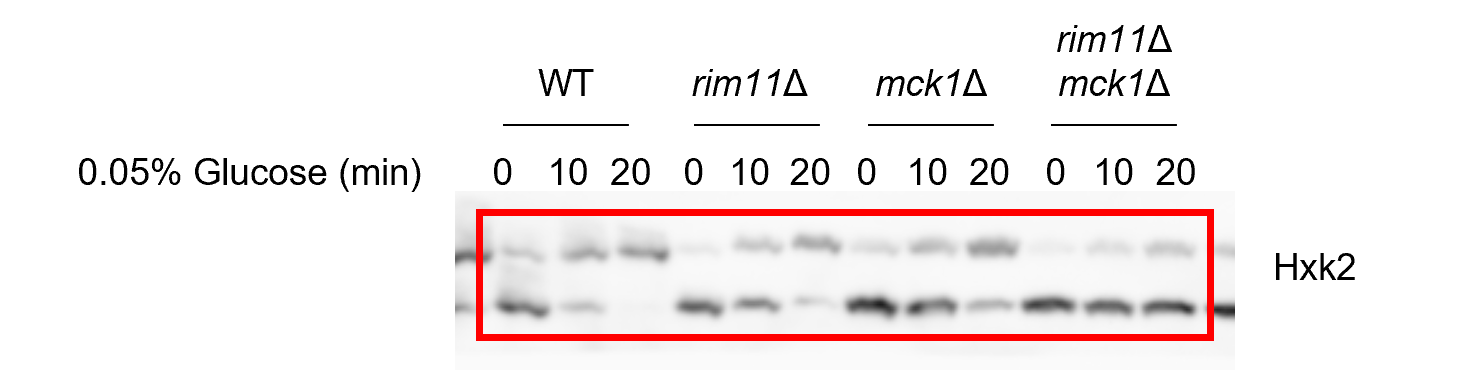

Supplement: Supplementary file 7 — Source data Fig. 3 [file 44319_2025_456_MOESM7_ESM.zip › Figure 3/Figure 3B/Figure 3B Hxk2.tif]

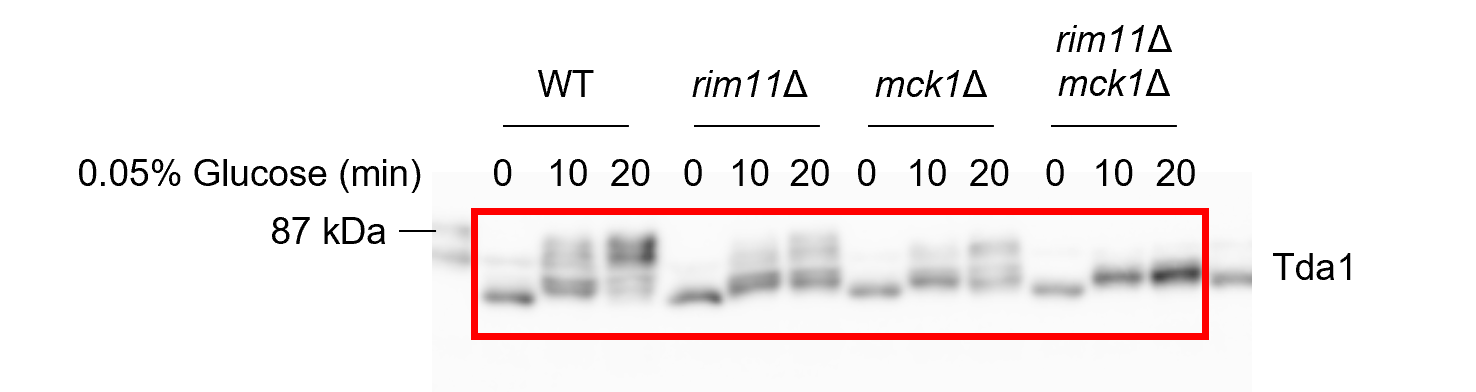

Supplement: Supplementary file 7 — Source data Fig. 3 [file 44319_2025_456_MOESM7_ESM.zip › Figure 3/Figure 3B/Figure 3B Tda1.tif]

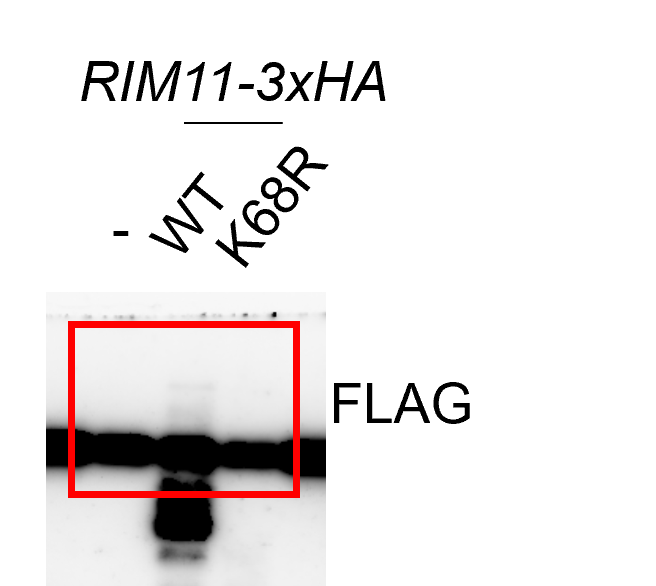

Supplement: Supplementary file 7 — Source data Fig. 3 [file 44319_2025_456_MOESM7_ESM.zip › Figure 3/Figure 3E/Figure 3E FLAG.tif]

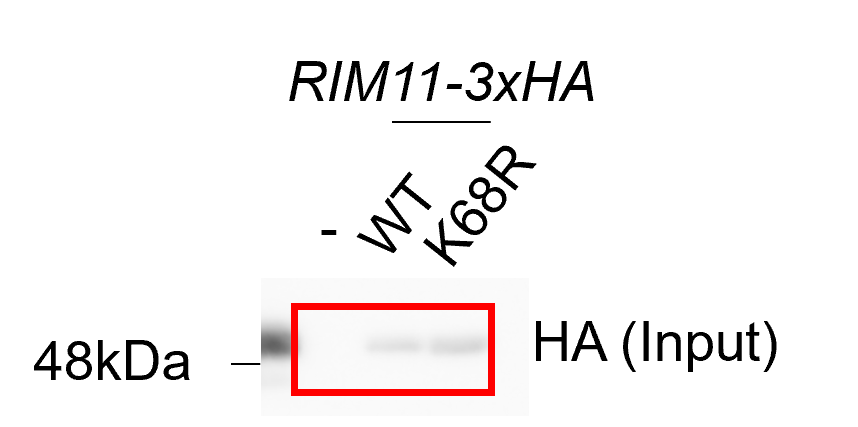

Supplement: Supplementary file 7 — Source data Fig. 3 [file 44319_2025_456_MOESM7_ESM.zip › Figure 3/Figure 3E/Figure 3E HA Input.tif]

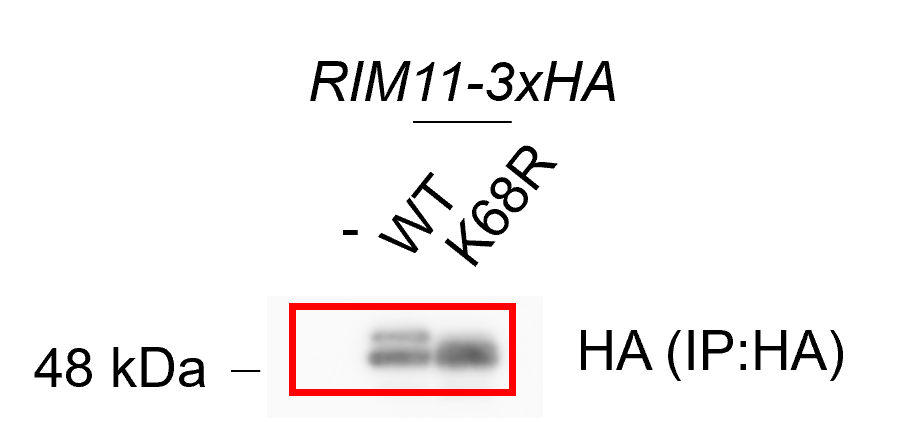

Supplement: Supplementary file 7 — Source data Fig. 3 [file 44319_2025_456_MOESM7_ESM.zip › Figure 3/Figure 3E/Figure 3E HA IP.tif]

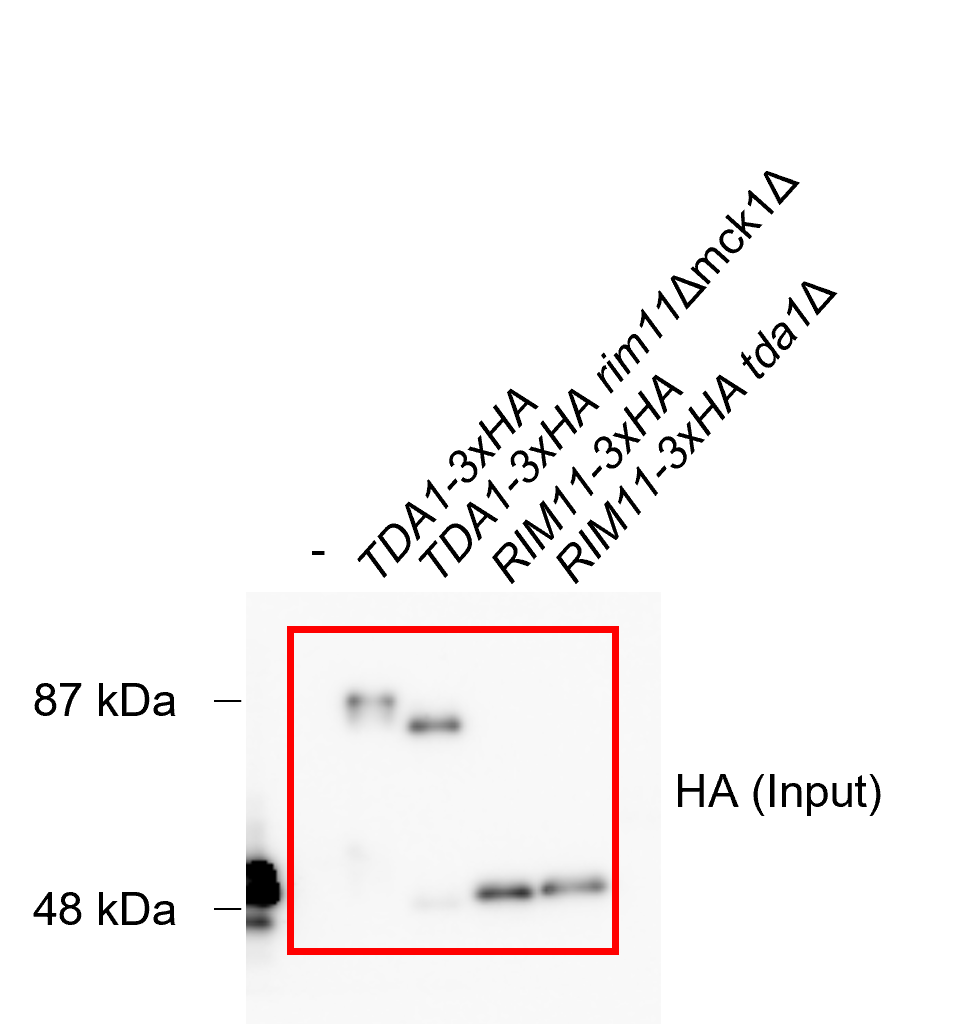

Supplement: Supplementary file 7 — Source data Fig. 3 [file 44319_2025_456_MOESM7_ESM.zip › Figure 3/Figure 3F/Figure 3F HA Input.tif]

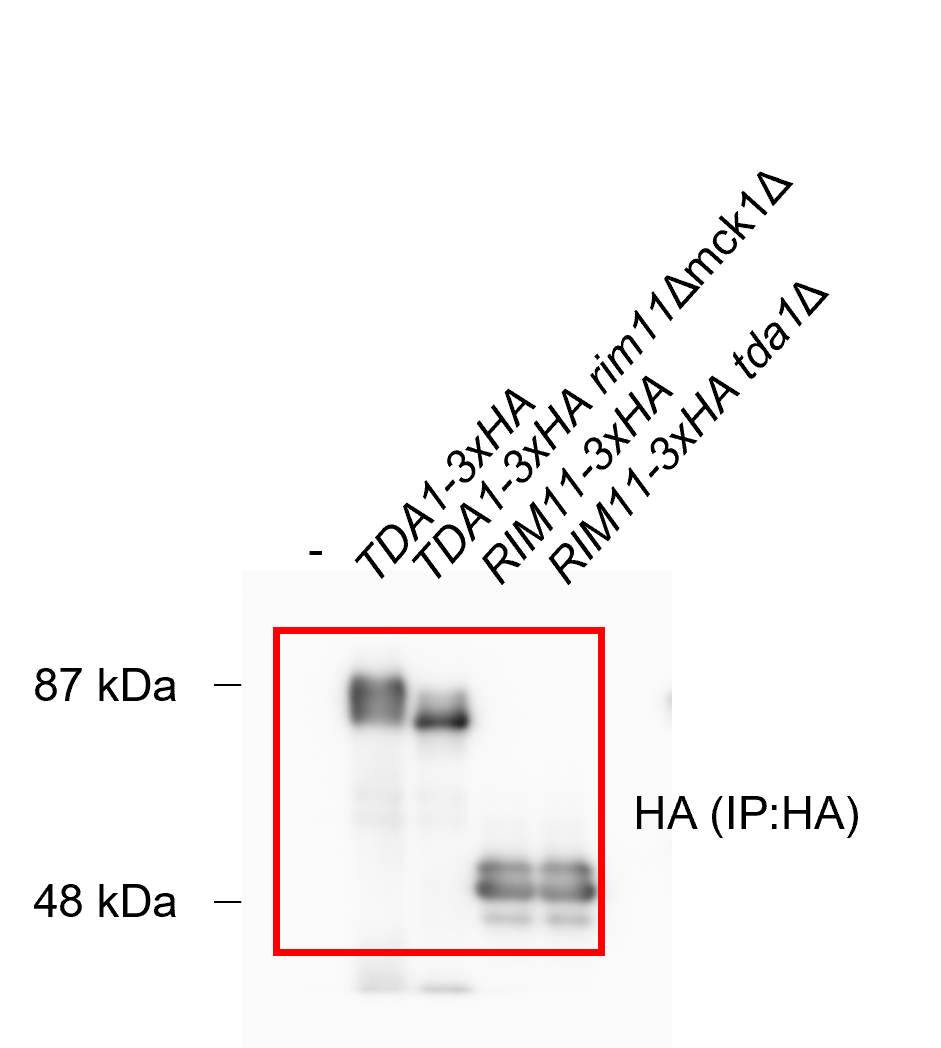

Supplement: Supplementary file 7 — Source data Fig. 3 [file 44319_2025_456_MOESM7_ESM.zip › Figure 3/Figure 3F/Figure 3F HA IP.tif]

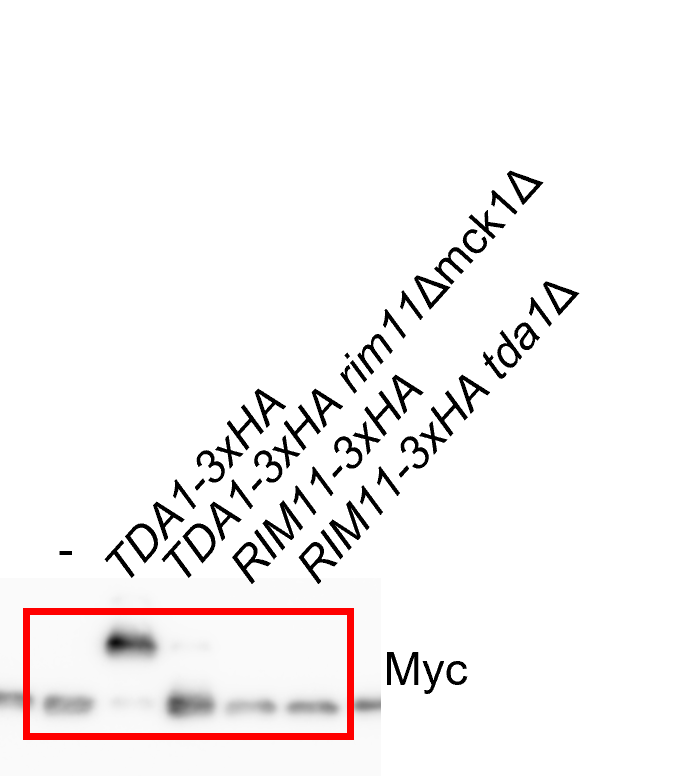

Supplement: Supplementary file 7 — Source data Fig. 3 [file 44319_2025_456_MOESM7_ESM.zip › Figure 3/Figure 3F/Figure 3F Myc.tif]

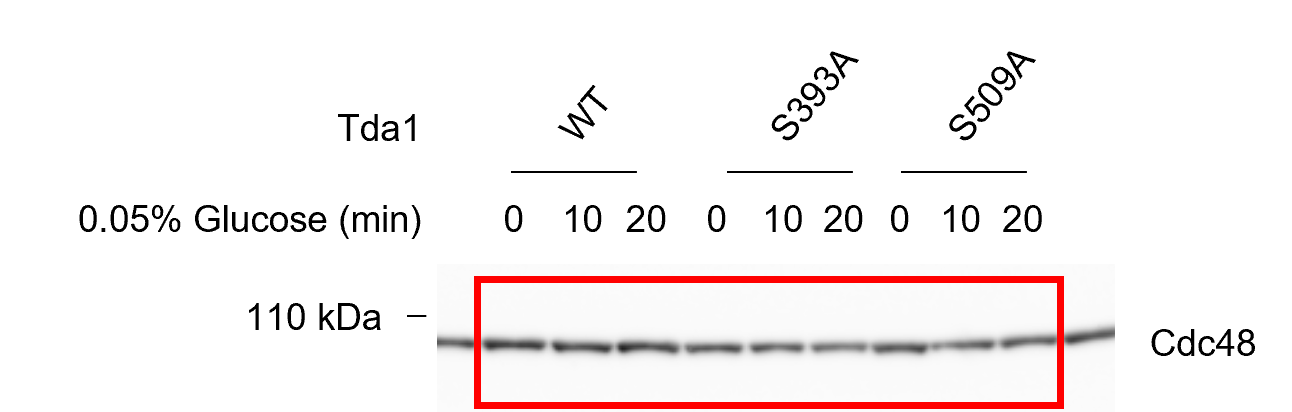

Supplement: Supplementary file 7 — Source data Fig. 3 [file 44319_2025_456_MOESM7_ESM.zip › Figure 3/Figure 3G/Figure 3G Cdc48.tif]

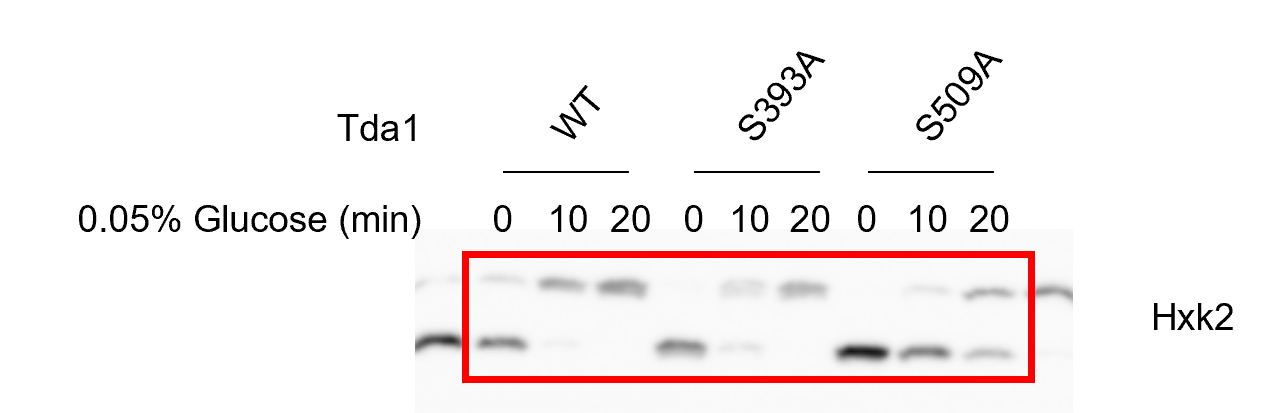

Supplement: Supplementary file 7 — Source data Fig. 3 [file 44319_2025_456_MOESM7_ESM.zip › Figure 3/Figure 3G/Figure 3G Hxk2.tif]

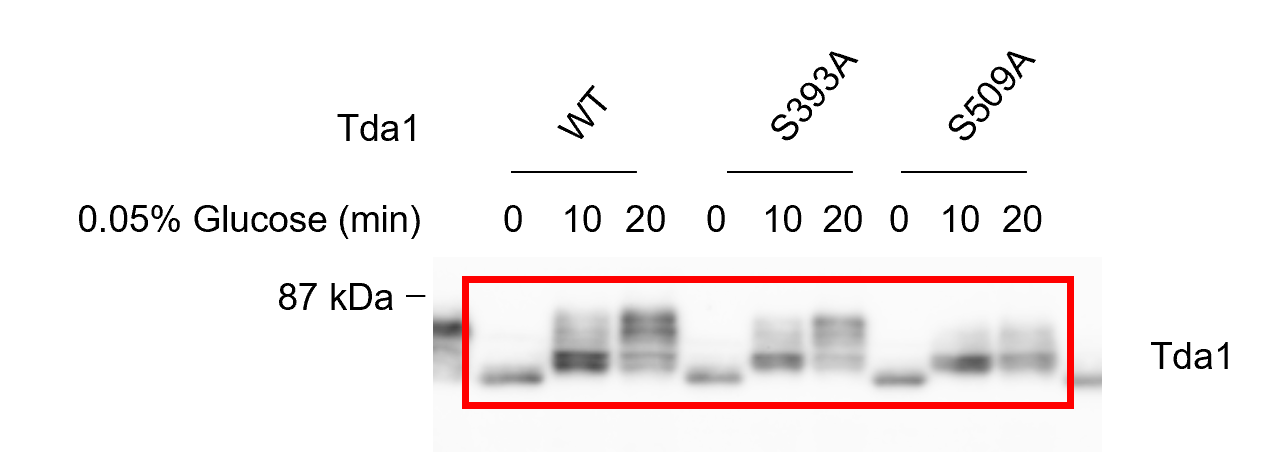

Supplement: Supplementary file 7 — Source data Fig. 3 [file 44319_2025_456_MOESM7_ESM.zip › Figure 3/Figure 3G/Figure 3G Tda1.tif]

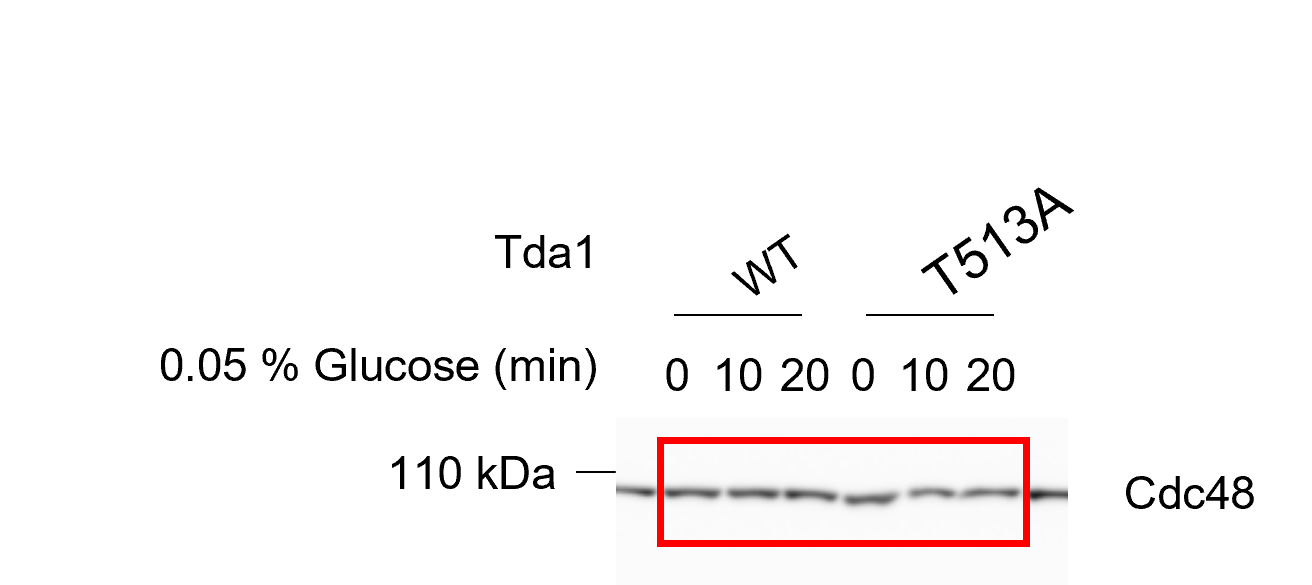

Supplement: Supplementary file 7 — Source data Fig. 3 [file 44319_2025_456_MOESM7_ESM.zip › Figure 3/Figure 3J/Figure 3J Cdc48.tif]

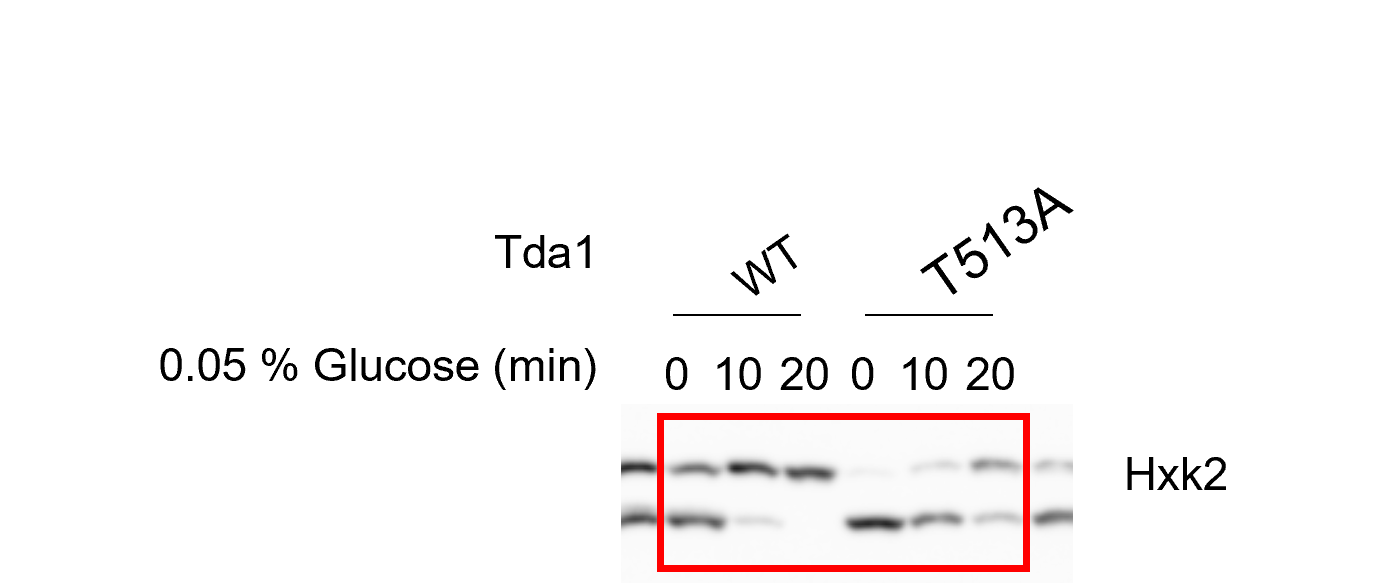

Supplement: Supplementary file 7 — Source data Fig. 3 [file 44319_2025_456_MOESM7_ESM.zip › Figure 3/Figure 3J/Figure 3J Hxk2.tif]

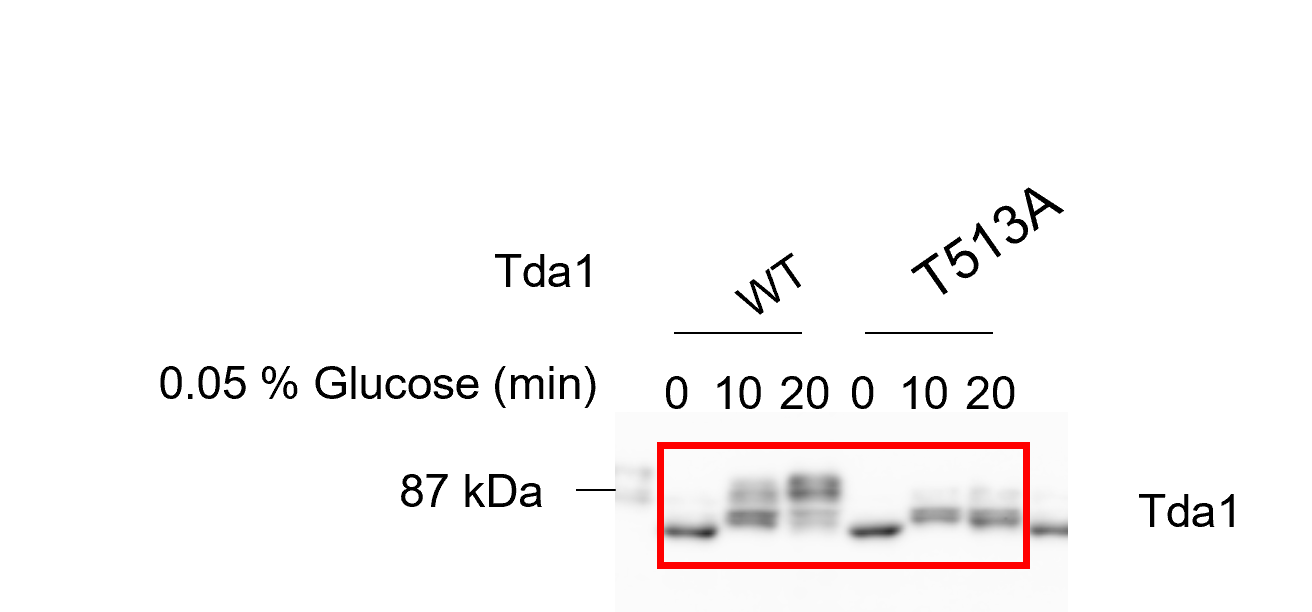

Supplement: Supplementary file 7 — Source data Fig. 3 [file 44319_2025_456_MOESM7_ESM.zip › Figure 3/Figure 3J/Figure 3J Tda1.tif]

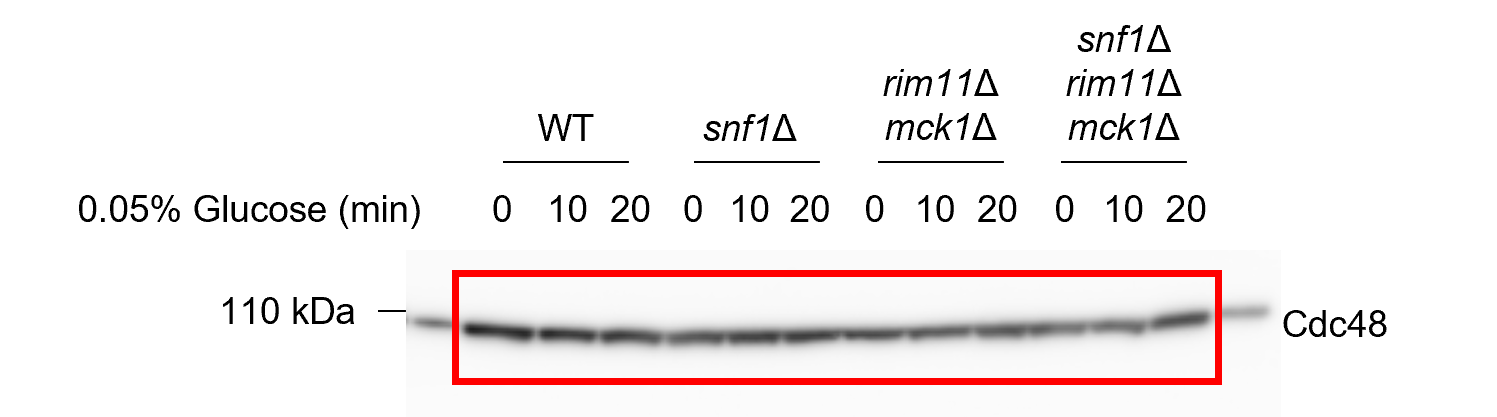

Supplement: Supplementary file 8 — Source data Fig. 4 [file 44319_2025_456_MOESM8_ESM.zip › Figure 4/Figure 4A/Figure 4A Cdc48.tif]

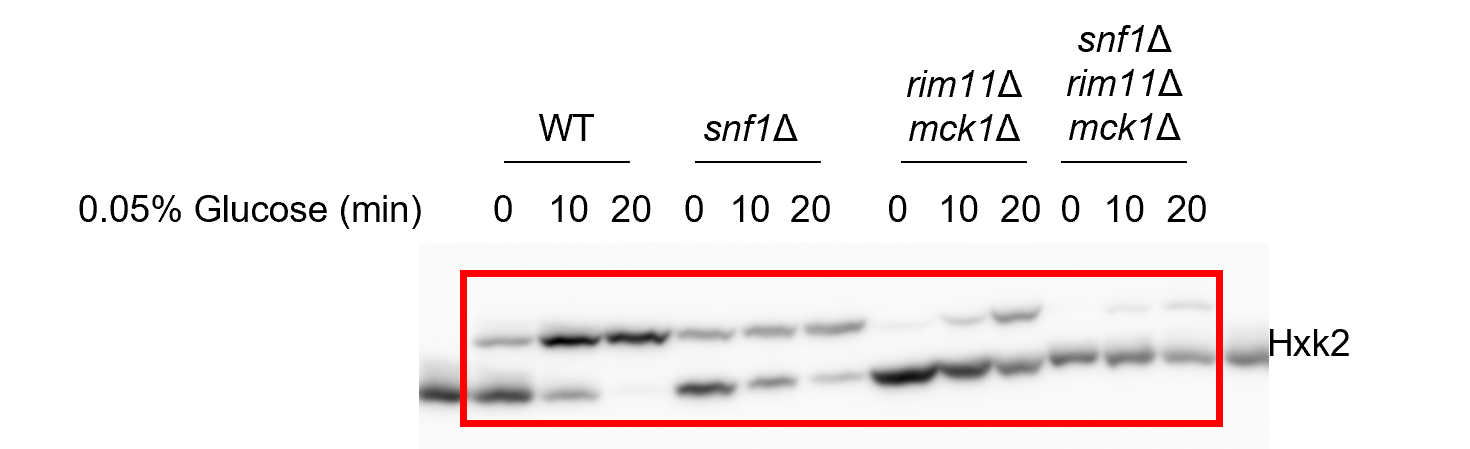

Supplement: Supplementary file 8 — Source data Fig. 4 [file 44319_2025_456_MOESM8_ESM.zip › Figure 4/Figure 4A/Figure 4A Hxk2.tif]

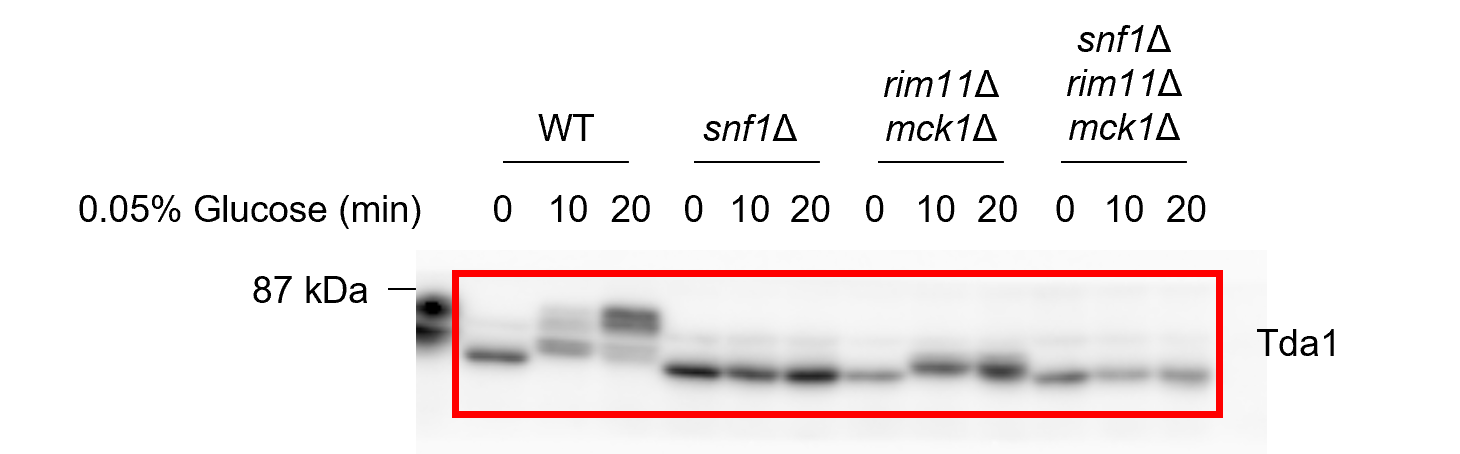

Supplement: Supplementary file 8 — Source data Fig. 4 [file 44319_2025_456_MOESM8_ESM.zip › Figure 4/Figure 4A/Figure 4A Tda1.tif]

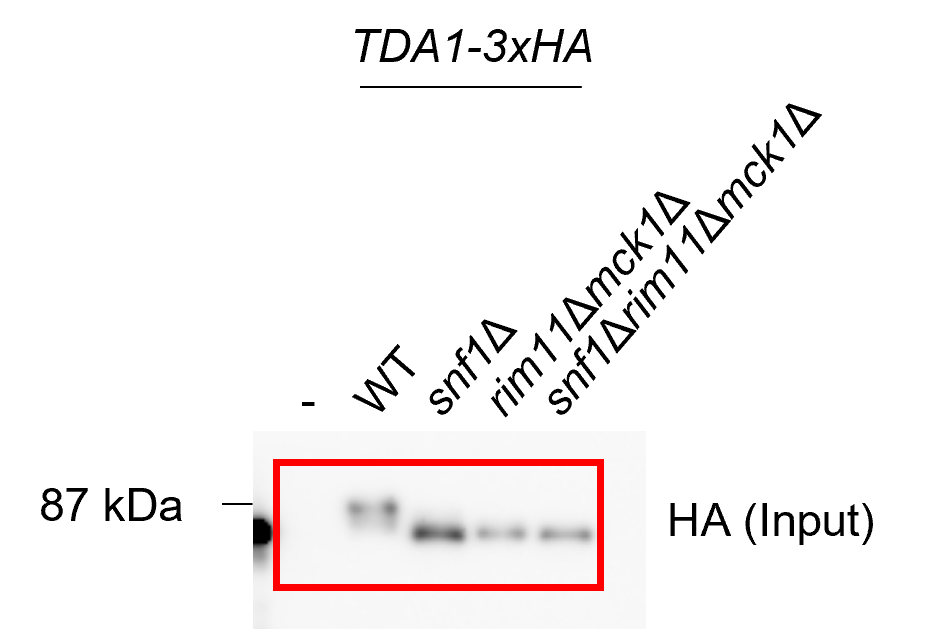

Supplement: Supplementary file 8 — Source data Fig. 4 [file 44319_2025_456_MOESM8_ESM.zip › Figure 4/Figure 4C/Figure 4C HA Input.tif]

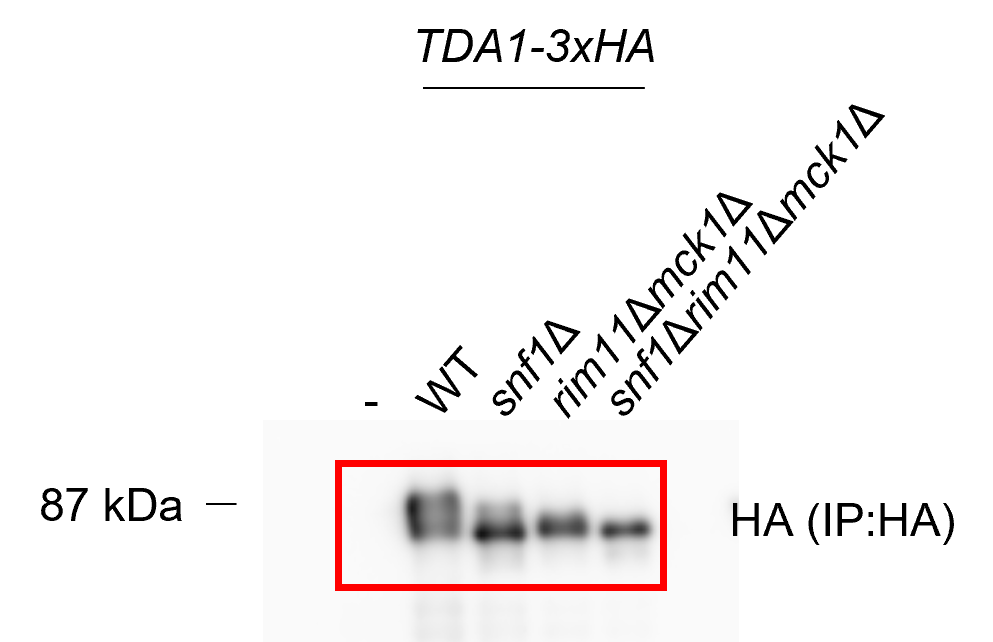

Supplement: Supplementary file 8 — Source data Fig. 4 [file 44319_2025_456_MOESM8_ESM.zip › Figure 4/Figure 4C/Figure 4C HA IP.tif]

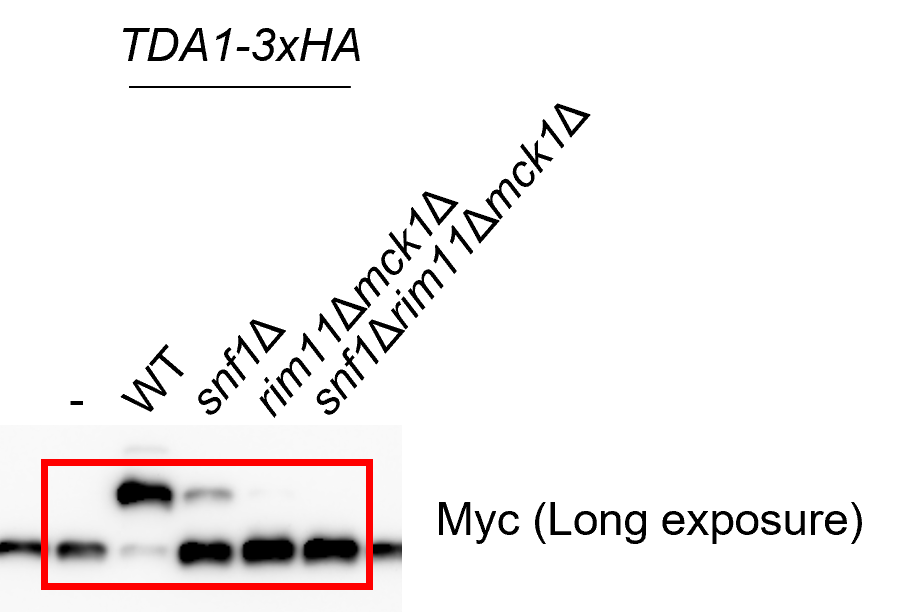

Supplement: Supplementary file 8 — Source data Fig. 4 [file 44319_2025_456_MOESM8_ESM.zip › Figure 4/Figure 4C/Figure 4C Myc(Long exposure).tif]

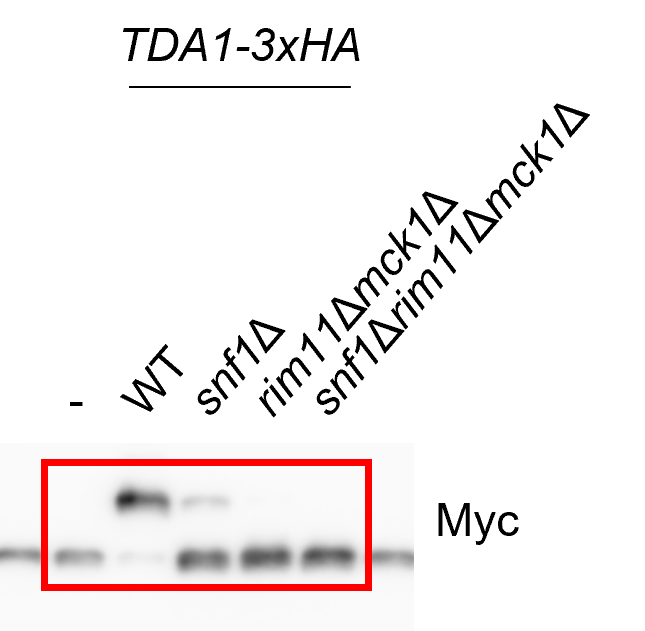

Supplement: Supplementary file 8 — Source data Fig. 4 [file 44319_2025_456_MOESM8_ESM.zip › Figure 4/Figure 4C/Figure 4C Myc.tif]

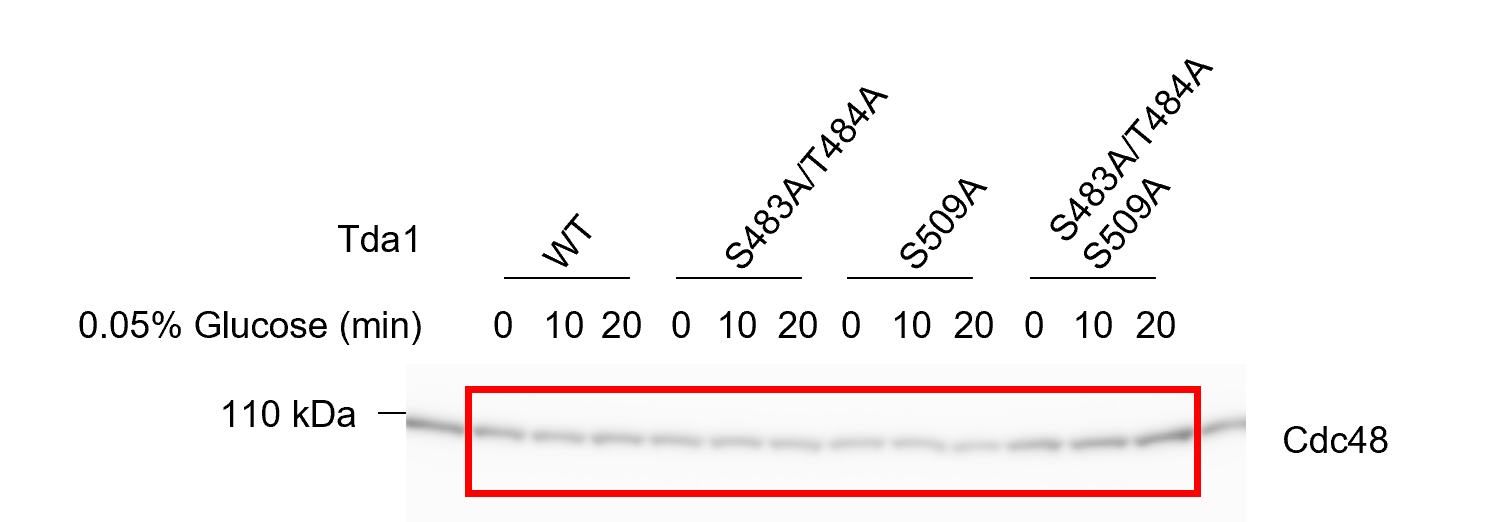

Supplement: Supplementary file 8 — Source data Fig. 4 [file 44319_2025_456_MOESM8_ESM.zip › Figure 4/Figure 4D/Figure 4D Cdc48.tif]

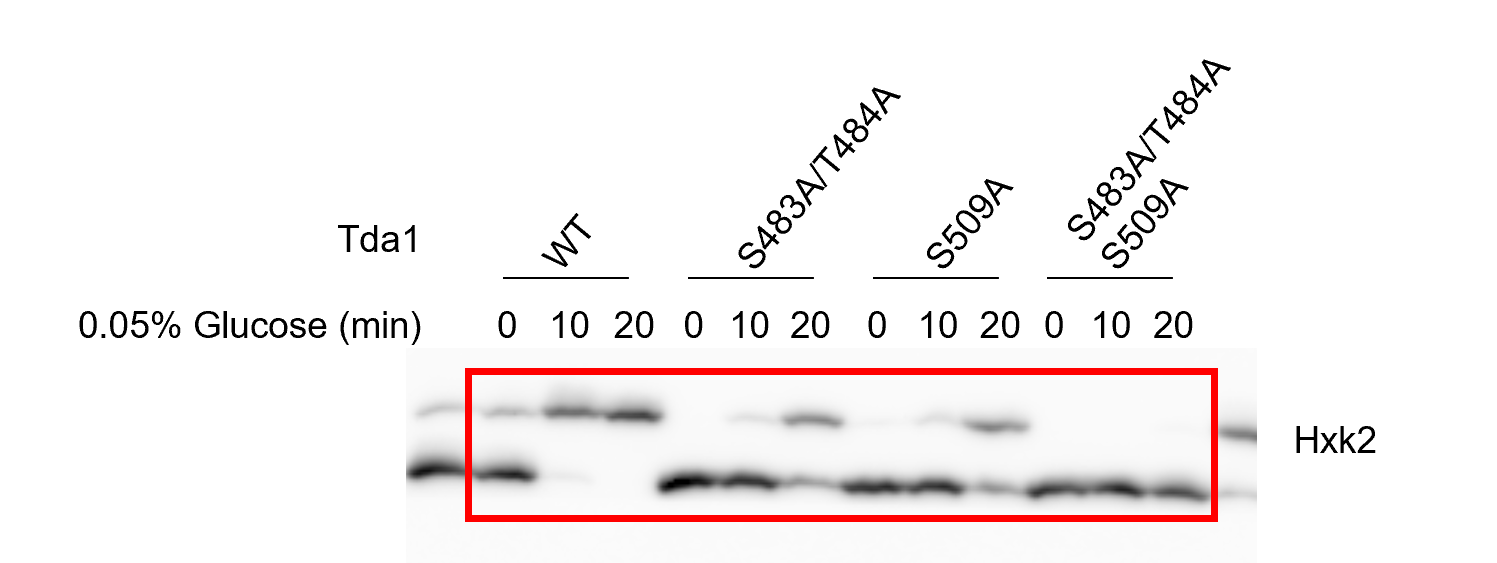

Supplement: Supplementary file 8 — Source data Fig. 4 [file 44319_2025_456_MOESM8_ESM.zip › Figure 4/Figure 4D/Figure 4D Hxk2.tif]

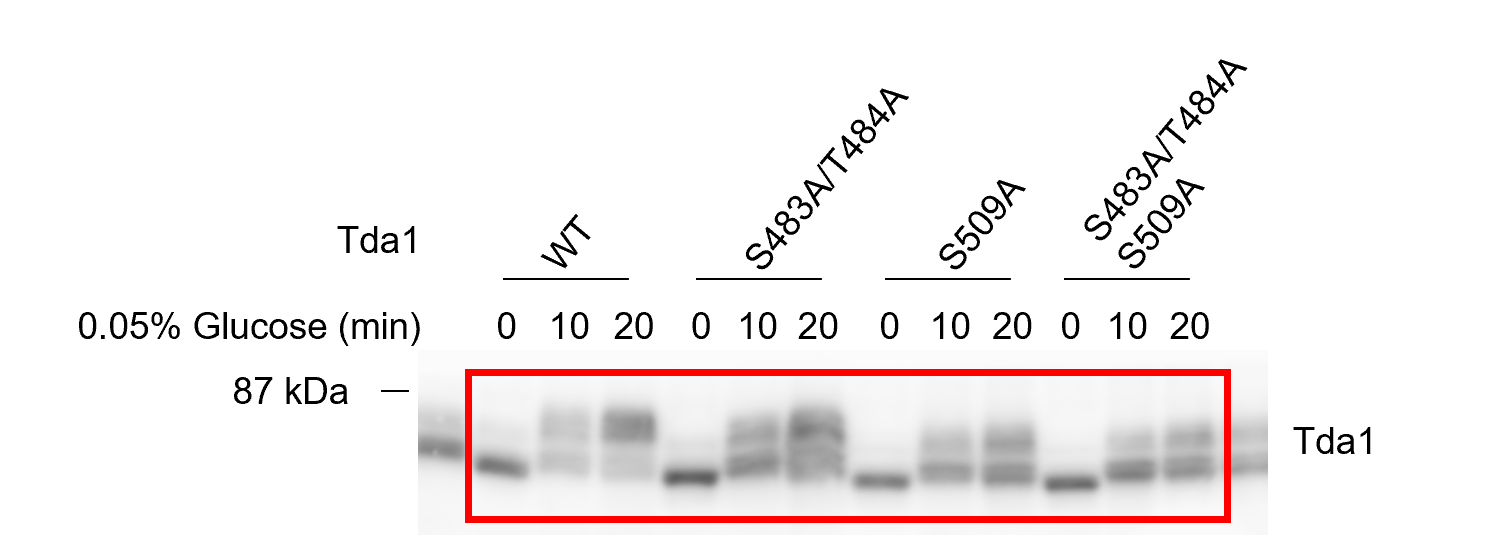

Supplement: Supplementary file 8 — Source data Fig. 4 [file 44319_2025_456_MOESM8_ESM.zip › Figure 4/Figure 4D/Figure 4D Tda1.tif]

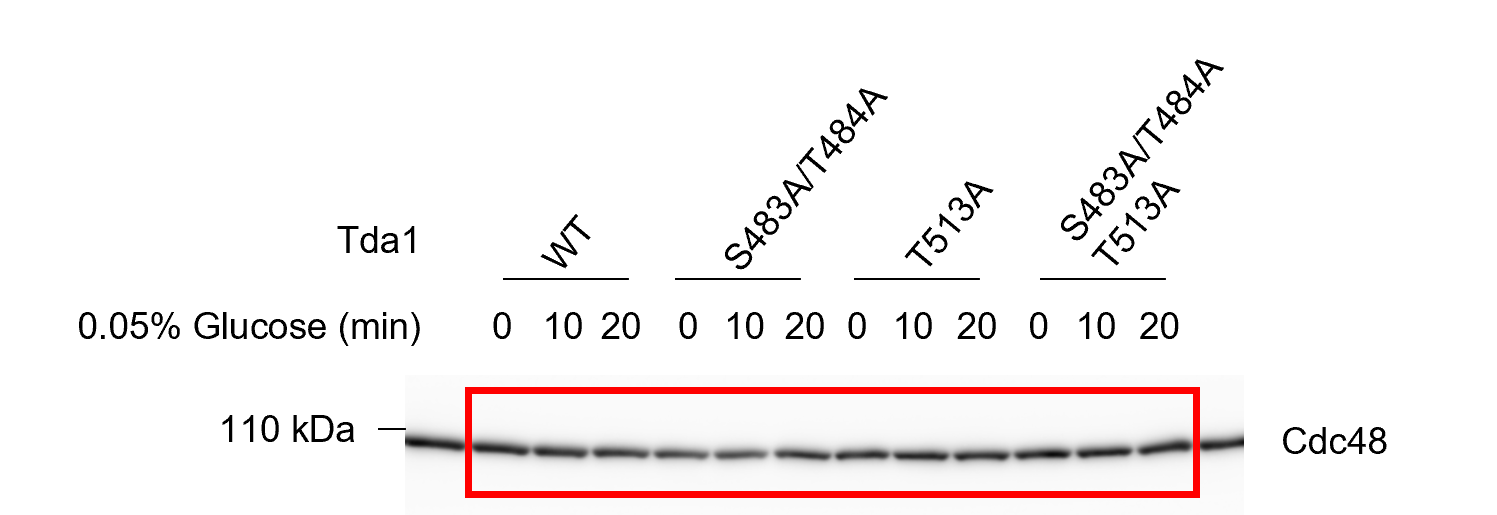

Supplement: Supplementary file 8 — Source data Fig. 4 [file 44319_2025_456_MOESM8_ESM.zip › Figure 4/Figure 4F/Figure 4F Cdc48.tif]

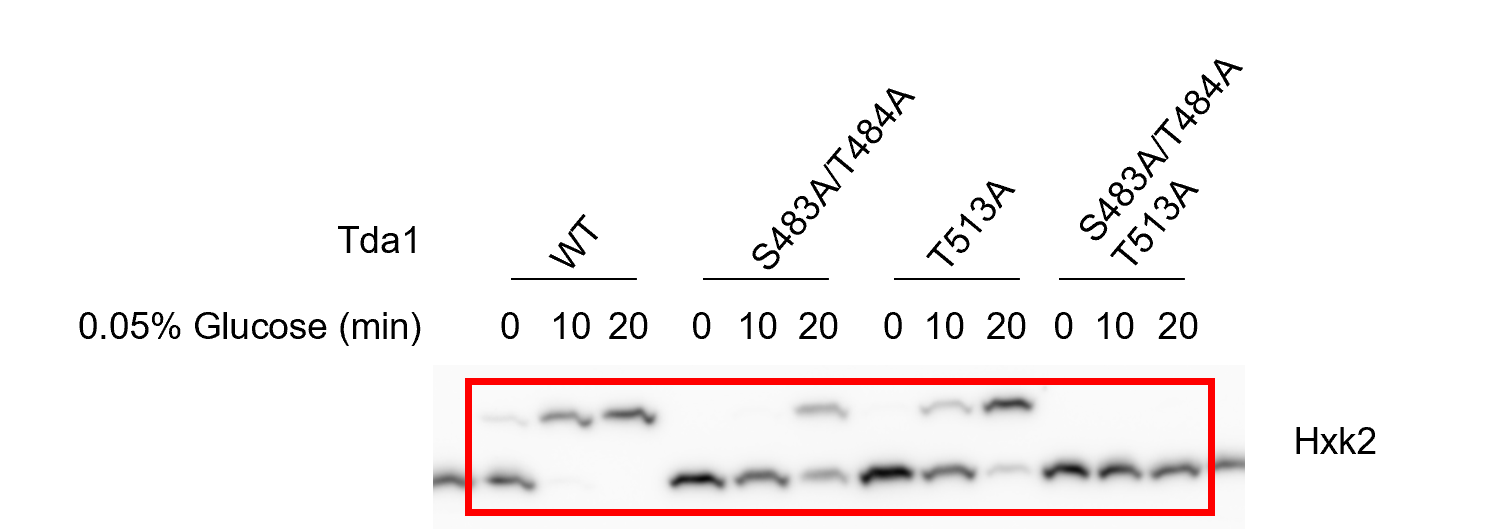

Supplement: Supplementary file 8 — Source data Fig. 4 [file 44319_2025_456_MOESM8_ESM.zip › Figure 4/Figure 4F/Figure 4F Hxk2.tif]

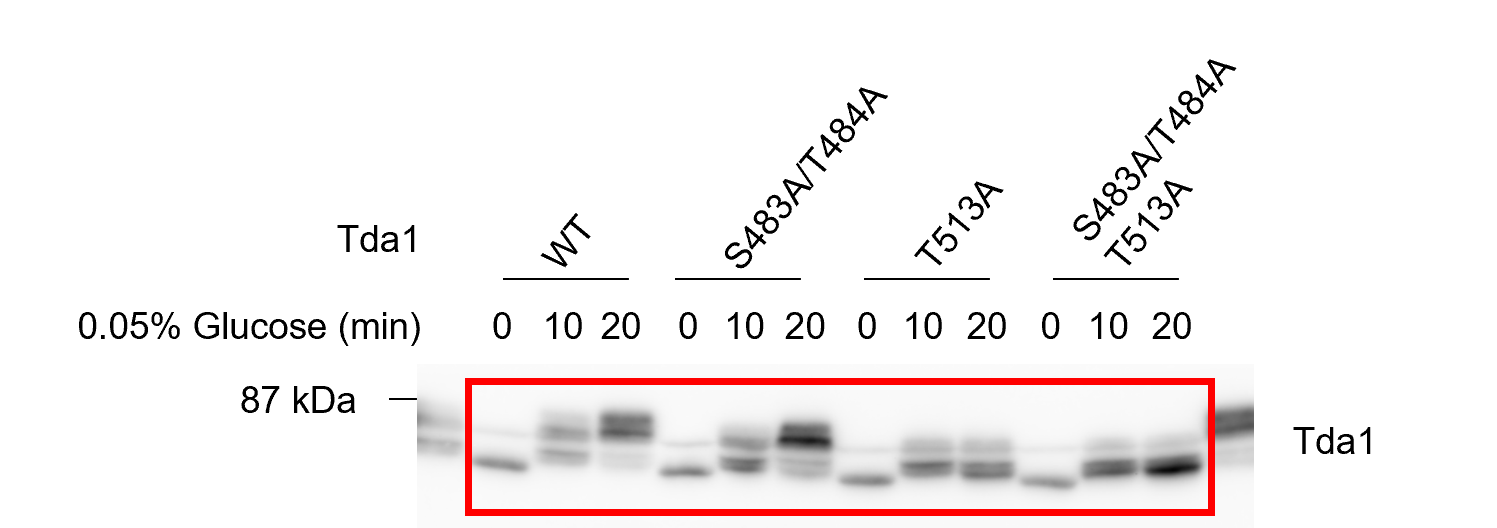

Supplement: Supplementary file 8 — Source data Fig. 4 [file 44319_2025_456_MOESM8_ESM.zip › Figure 4/Figure 4F/Figure 4F Tda1.tif]

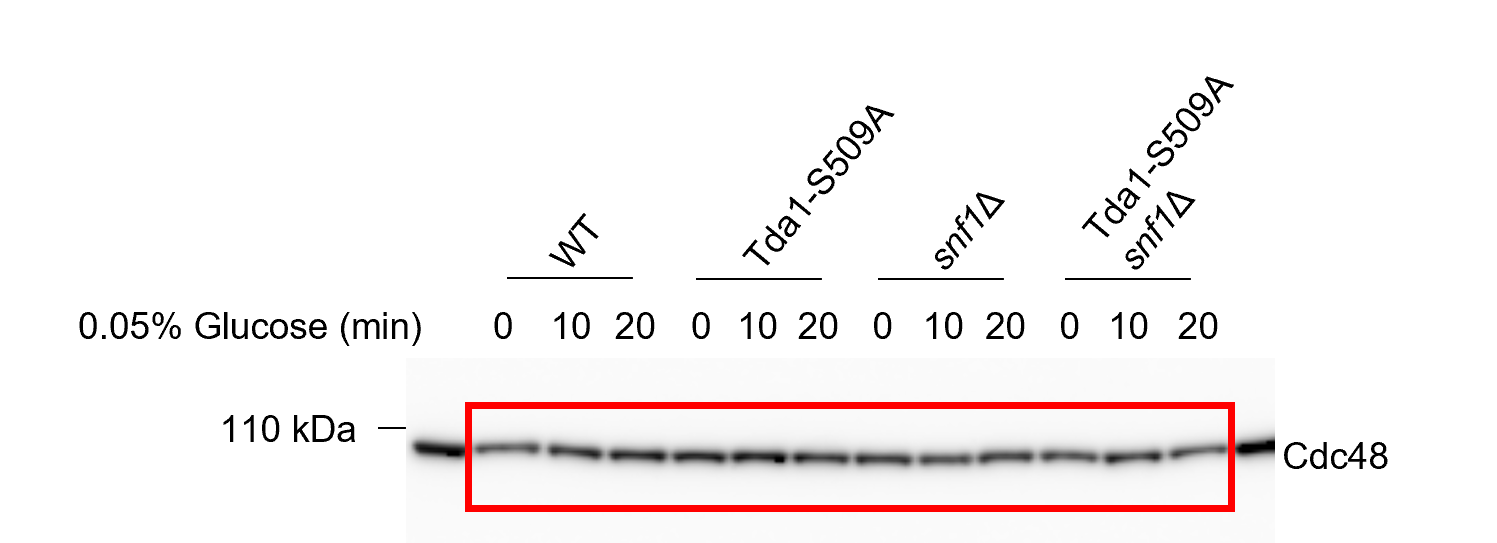

Supplement: Supplementary file 9 — Source data Fig. 5 [file 44319_2025_456_MOESM9_ESM.zip › Figure 5/Figure 5A/Figure 5A Cdc48.tif]

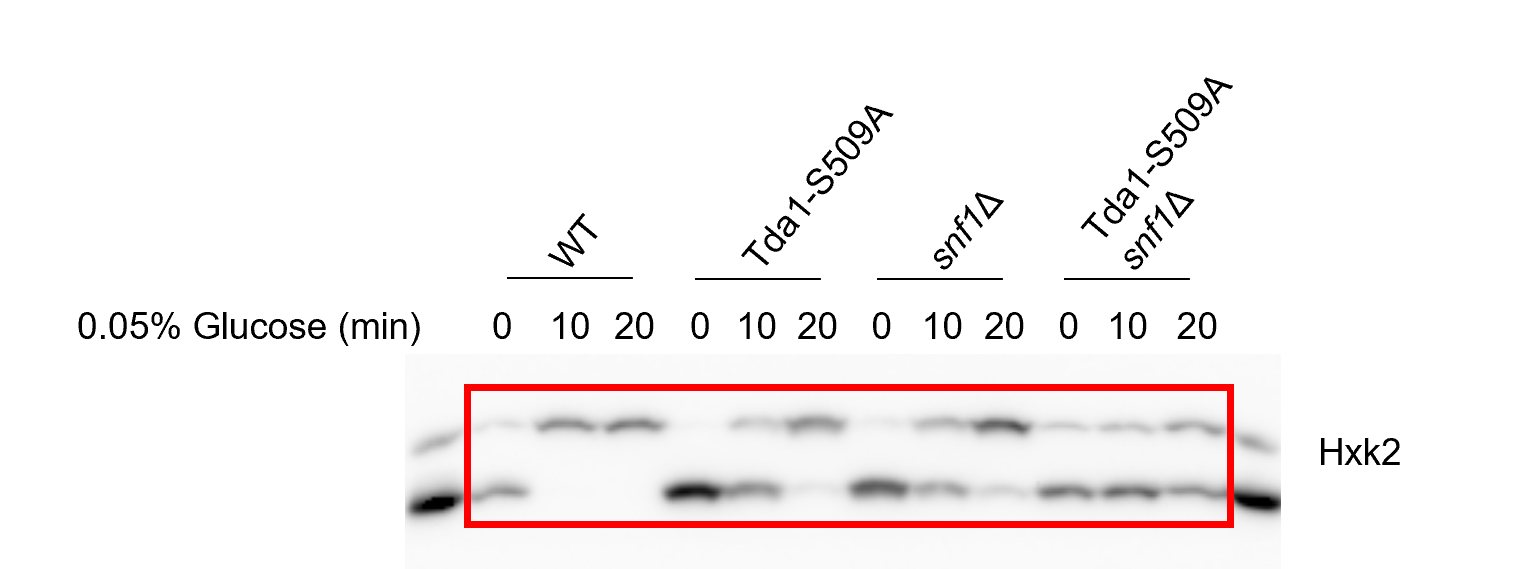

Supplement: Supplementary file 9 — Source data Fig. 5 [file 44319_2025_456_MOESM9_ESM.zip › Figure 5/Figure 5A/Figure 5A Hxk2.tif]

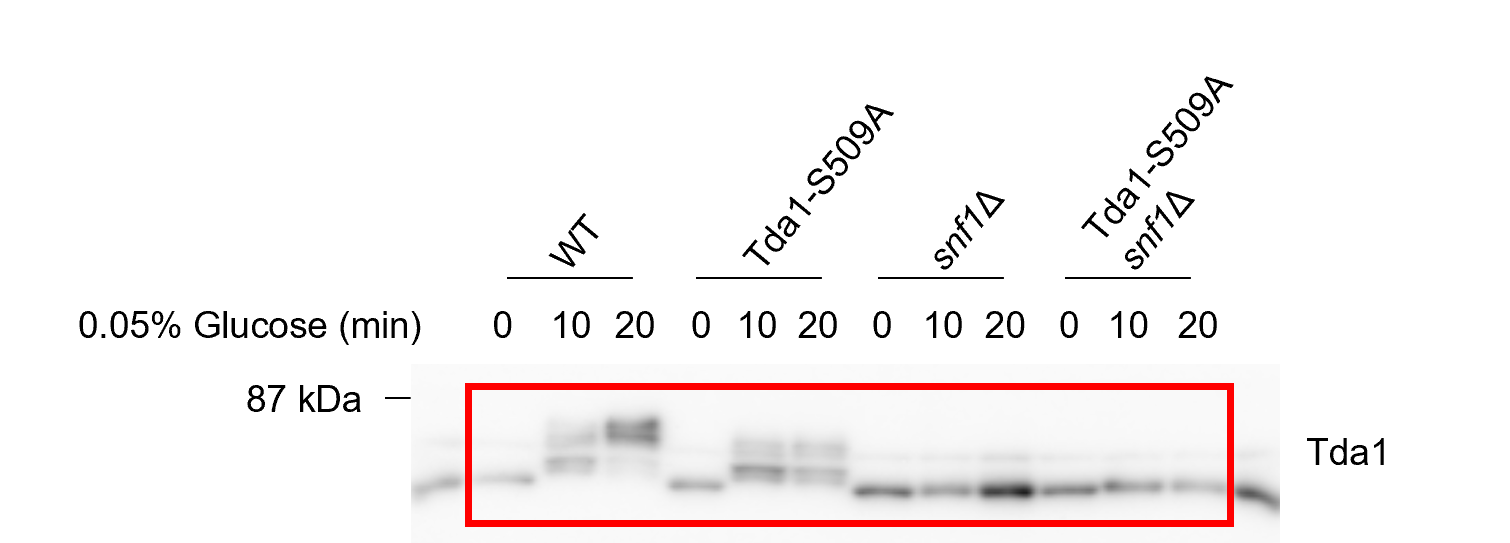

Supplement: Supplementary file 9 — Source data Fig. 5 [file 44319_2025_456_MOESM9_ESM.zip › Figure 5/Figure 5A/Figure 5A Tda1.tif]

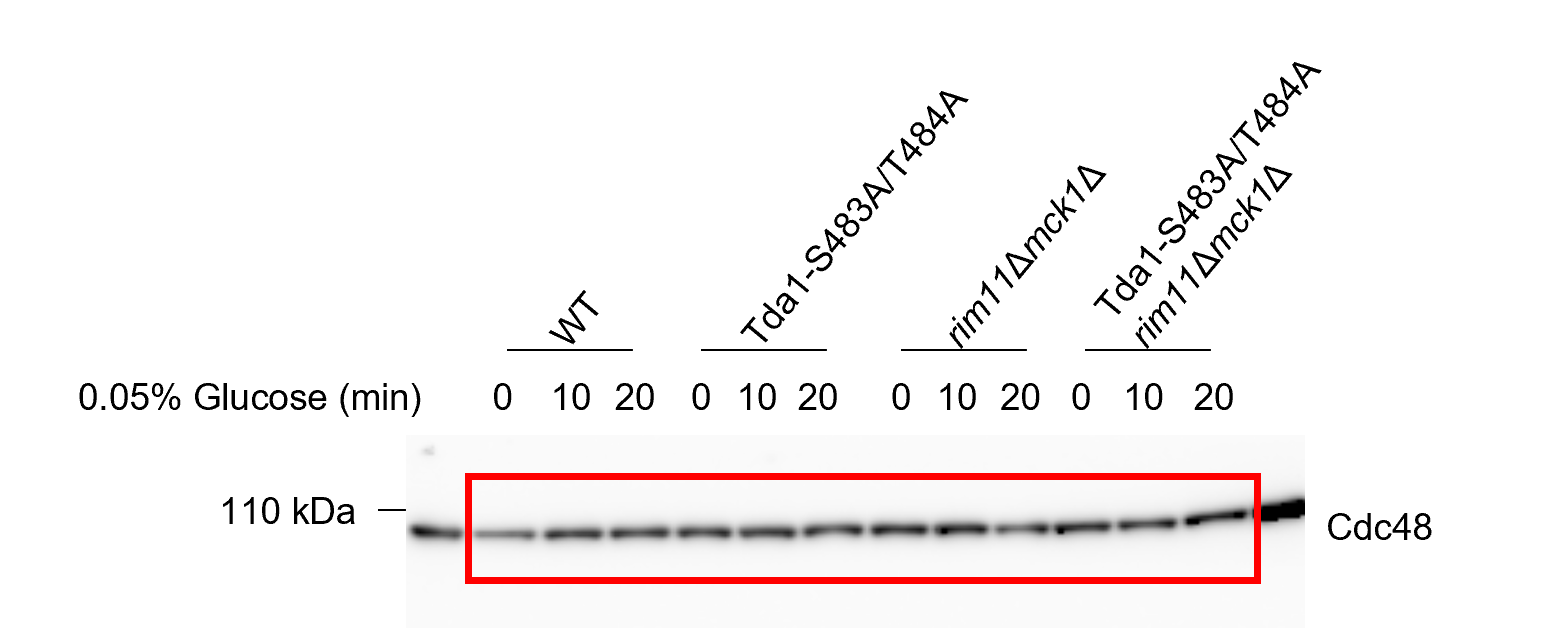

Supplement: Supplementary file 9 — Source data Fig. 5 [file 44319_2025_456_MOESM9_ESM.zip › Figure 5/Figure 5C/Figure 5C Cdc48.tif]

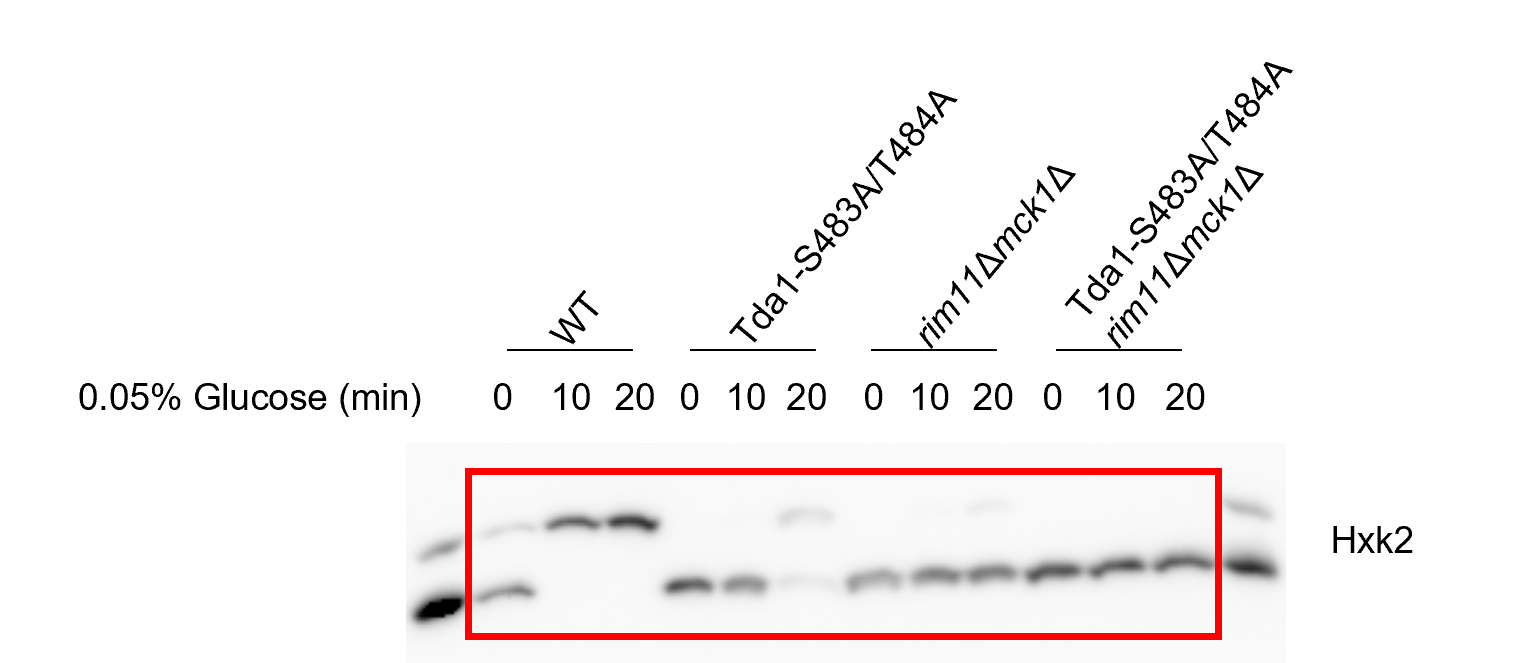

Supplement: Supplementary file 9 — Source data Fig. 5 [file 44319_2025_456_MOESM9_ESM.zip › Figure 5/Figure 5C/Figure 5C Hxk2.tif]

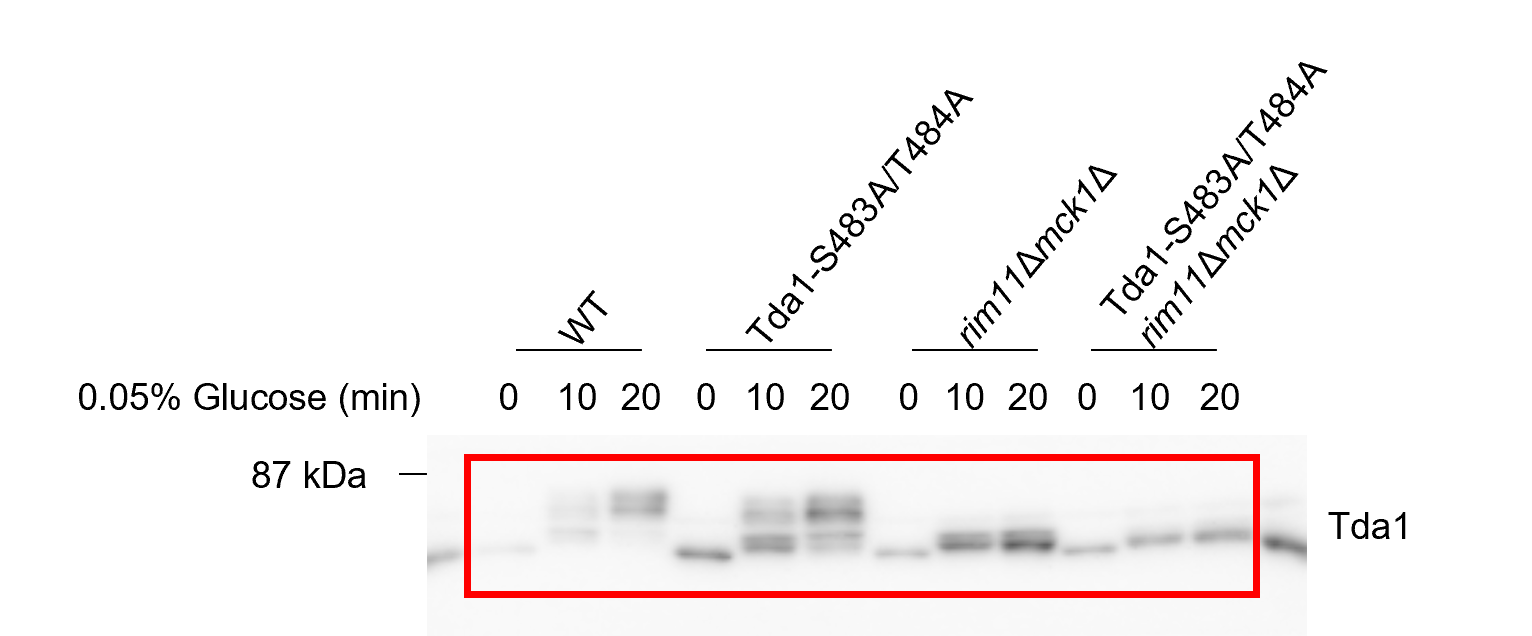

Supplement: Supplementary file 9 — Source data Fig. 5 [file 44319_2025_456_MOESM9_ESM.zip › Figure 5/Figure 5C/Figure 5C Tda1.tif]

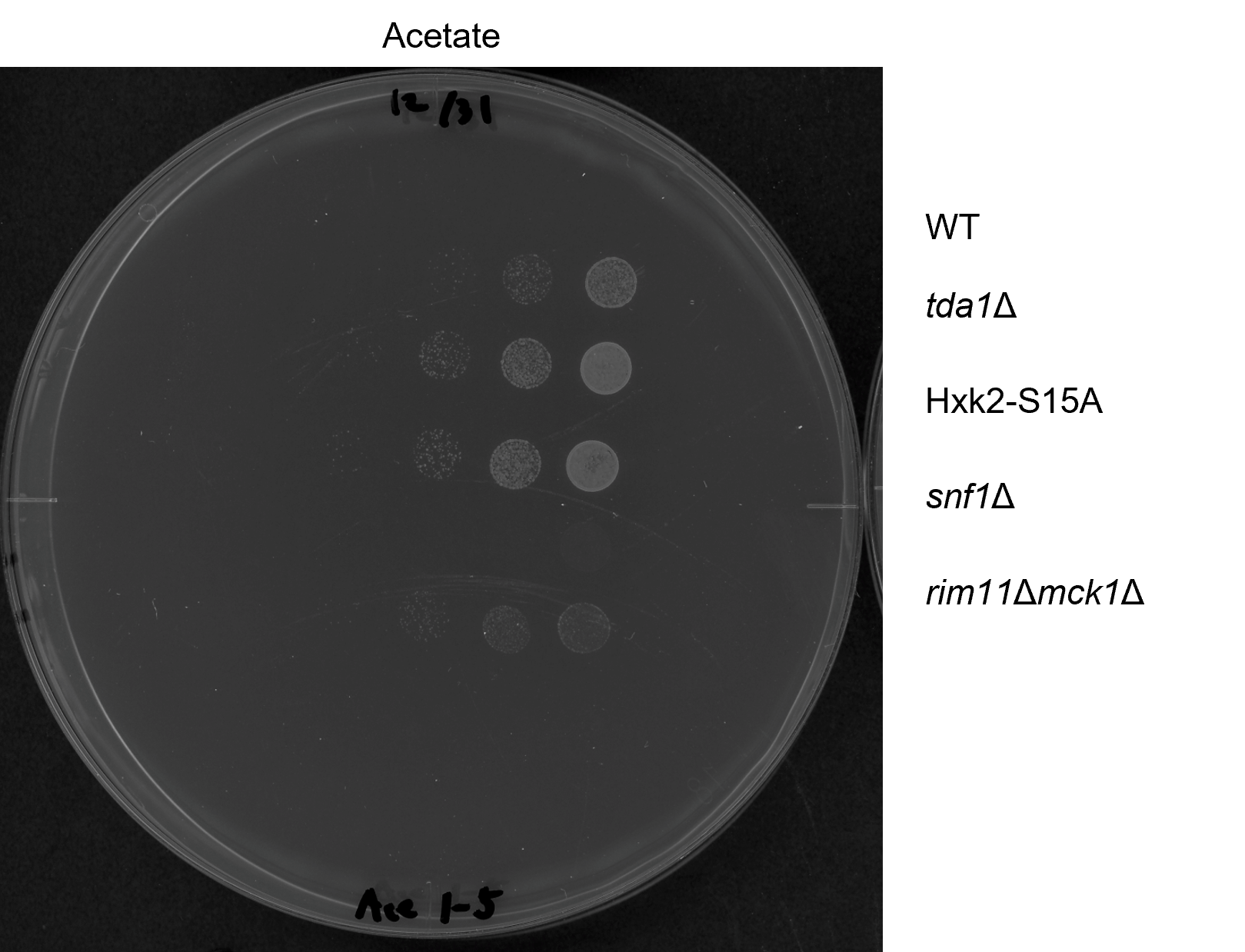

Supplement: Supplementary file 10 — Source data Fig. 6 [file 44319_2025_456_MOESM10_ESM.zip › Figure 6/Figure 6A/Figure 6A Acetate.tif]

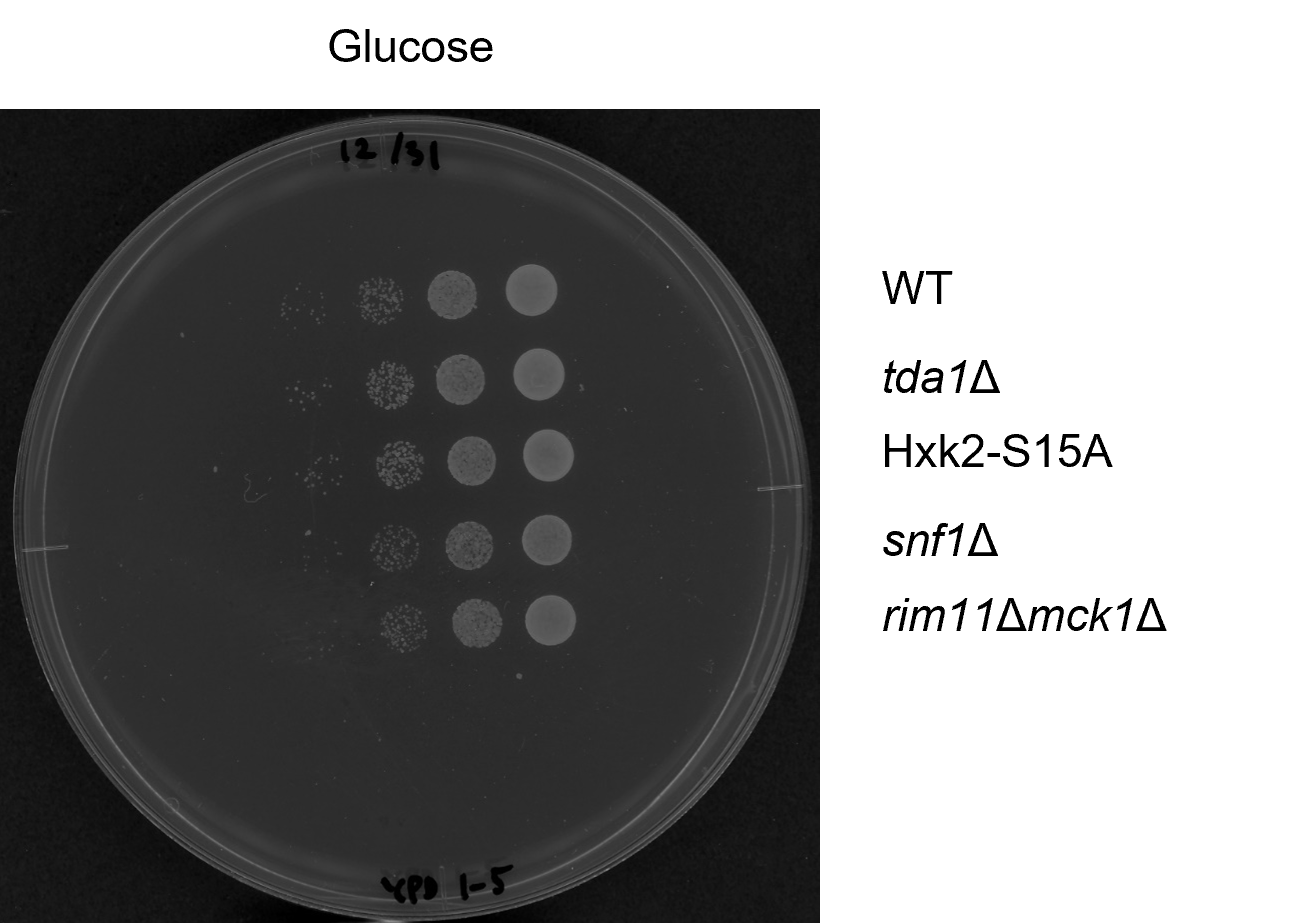

Supplement: Supplementary file 10 — Source data Fig. 6 [file 44319_2025_456_MOESM10_ESM.zip › Figure 6/Figure 6A/Figure 6A Glucose.tif]

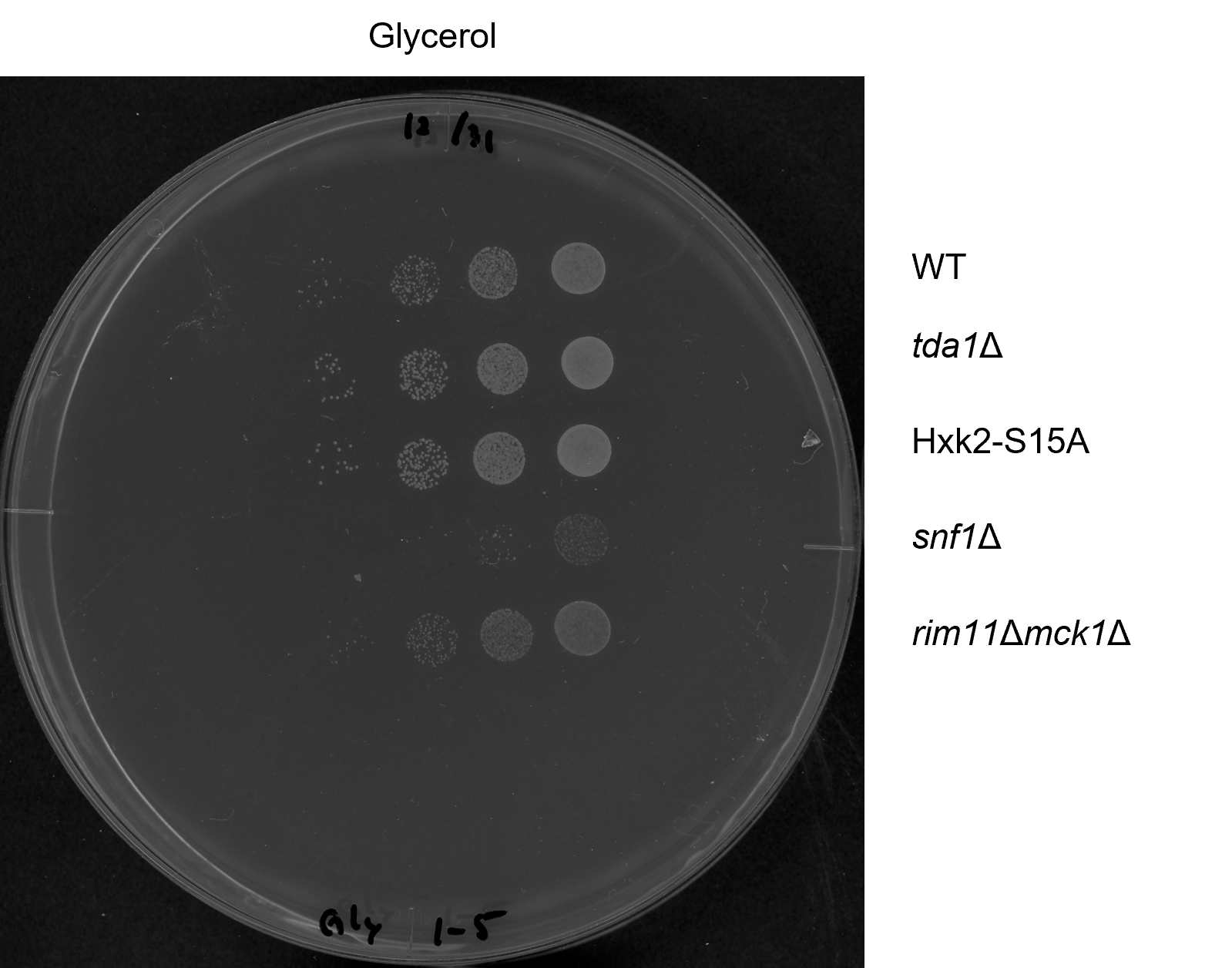

Supplement: Supplementary file 10 — Source data Fig. 6 [file 44319_2025_456_MOESM10_ESM.zip › Figure 6/Figure 6A/Figure 6A Glycerol.tif]
