# Supplementary material for: Snf1 and yeast GSK3-β activates Tda1 to suppress glucose starvation signaling
Source: EMBO Rep. 2025 Apr 24;26(11):2910–30. doi: 10.1038/s44319-025-00456-y (PMC12152124; doi:10.1038/s44319-025-00456-y)
Supplement: Supplementary file 10 — Source data Fig. 6 [file 44319_2025_456_MOESM10_ESM.zip › Figure 6/Figure 6C-E/Figure 6C-E.docx]

The RNA-seq data are deposited in NCBI GEO under the accession GSE290495. The data can be accessed using the token onunwykoxhabncl at the following page:
<https://www.ncbi.nlm.nih.gov/geo/query/acc.cgi?acc=GSE290495>.
